# Supplementary material for: Untargeted Analysis of Serum Metabolomes in Dogs with Exocrine Pancreatic Insufficiency
Source: Animals (Basel). 2023 Jul 14;13(14):2313. doi: 10.3390/ani13142313 (PMC10376357; doi:10.3390/ani13142313)
Supplement: Supplementary file 1 [file animals-13-02313-s001.zip › animals-2380457-supplementary/Supplemental Info/File S3.pdf]

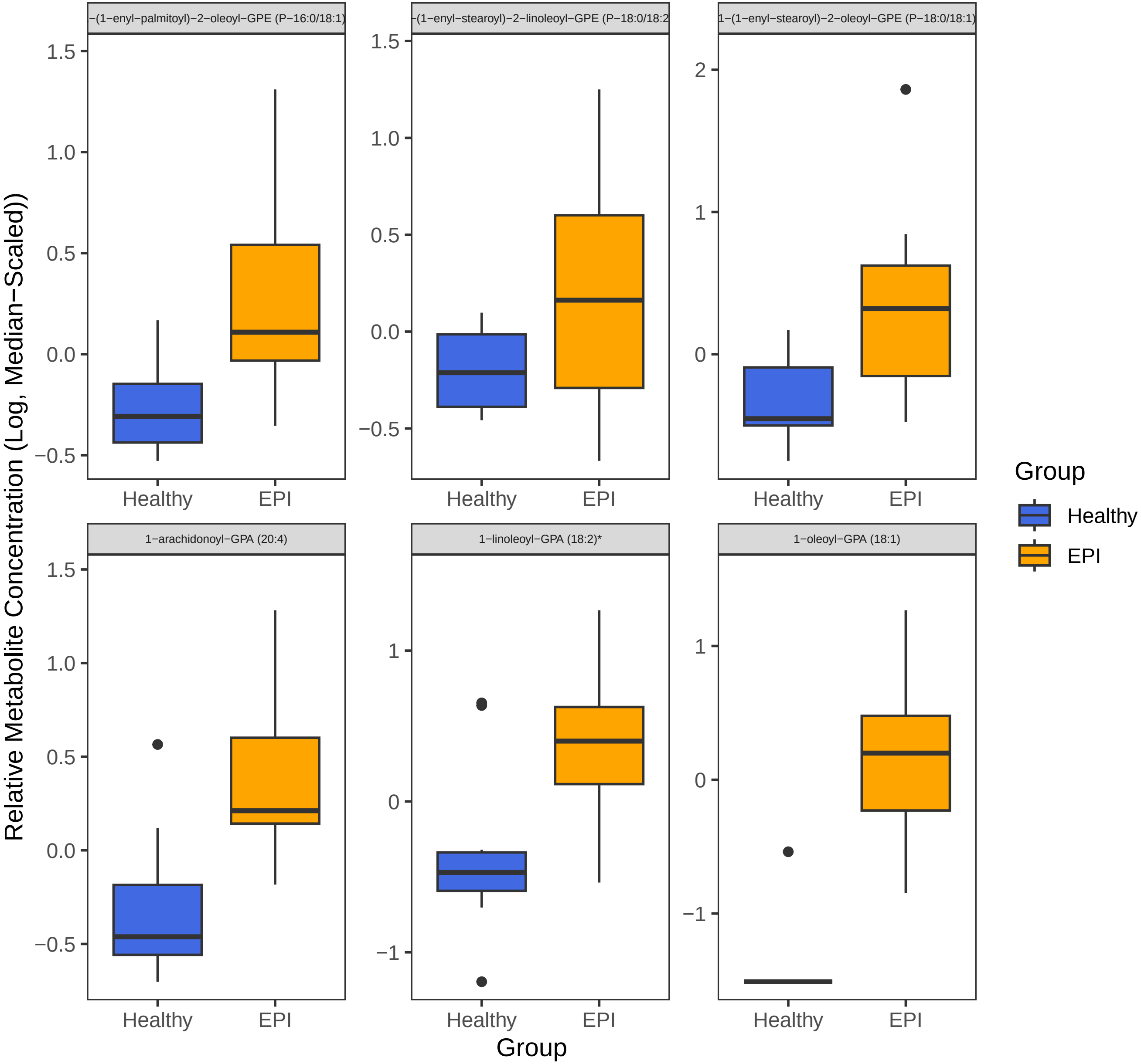

−(1−enyl−palmitoyl)−2−oleoyl−GPE (P−16:0/18:1)
-(1−enyl−stearoyl)−2−linoleoyl−GPE (P−18:0/18:2
1−(1−enyl−stearoyl)−2−oleoyl−GPE (P−18:0/18:1)
1−arachidonoyl−GPA (20:4)
1−linoleoyl−GPA (18:2)*
1−oleoyl−GPA (18:1)
Relative Metabolite Concentration (Log, Median−Scaled))
Healthy
EPI
Group
Group
Healthy
EPI

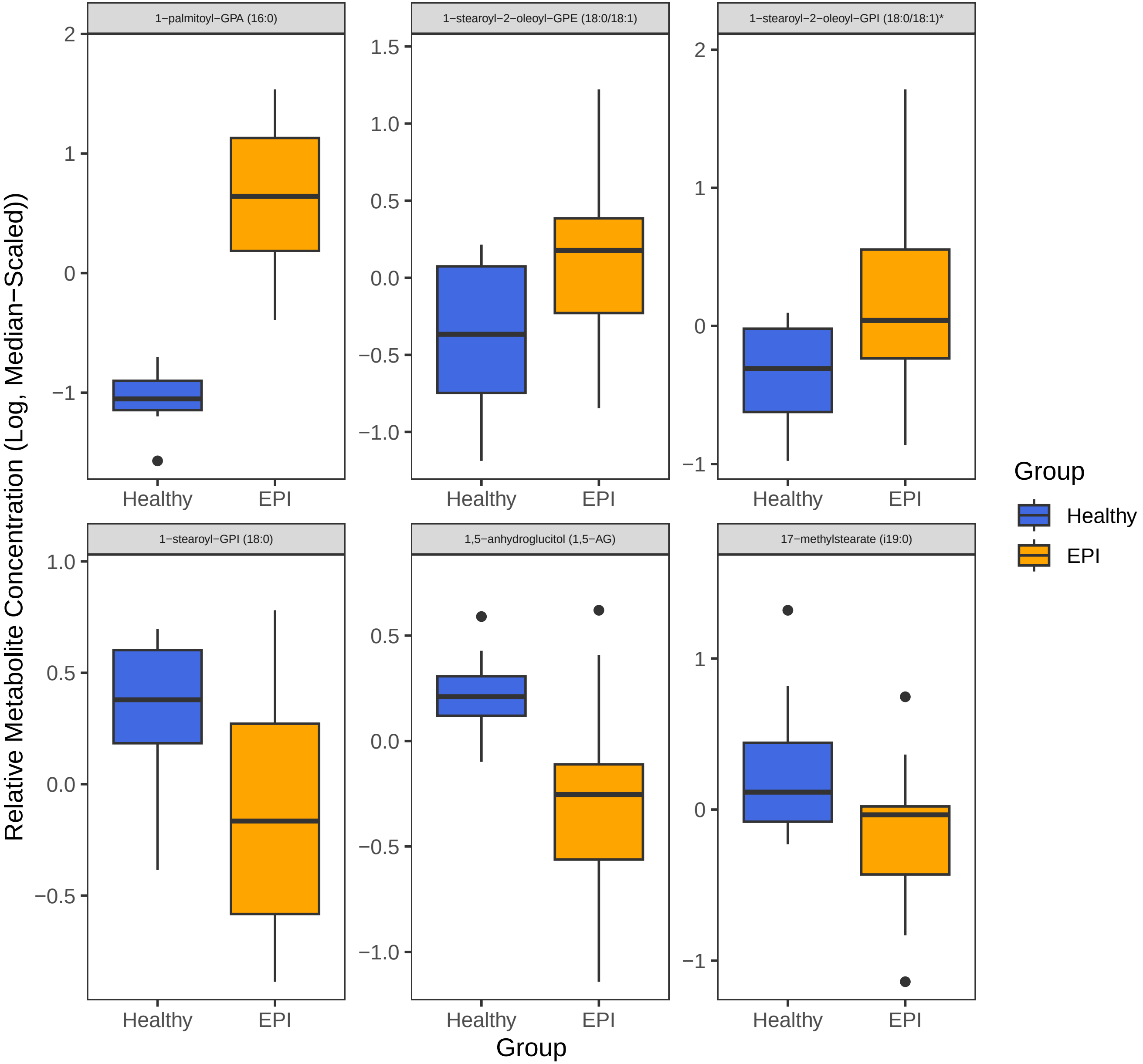
1−palmitoyl−GPA (16:0)
1−stearoyl−2−oleoyl−GPE (18:0/18:1)
1−stearoyl−2−oleoyl−GPI (18:0/18:1)*
1−stearoyl−GPI (18:0)
1,5−anhydroglucitol (1,5−AG)
17−methylstearate (i19:0)
Relative Metabolite Concentration (Log, Median−Scaled))
Group
Healthy
EPI
2
1
0
−1
1.5
1.0
0.5
0.0
−0.5
−1.0
Group
Healthy
EPI

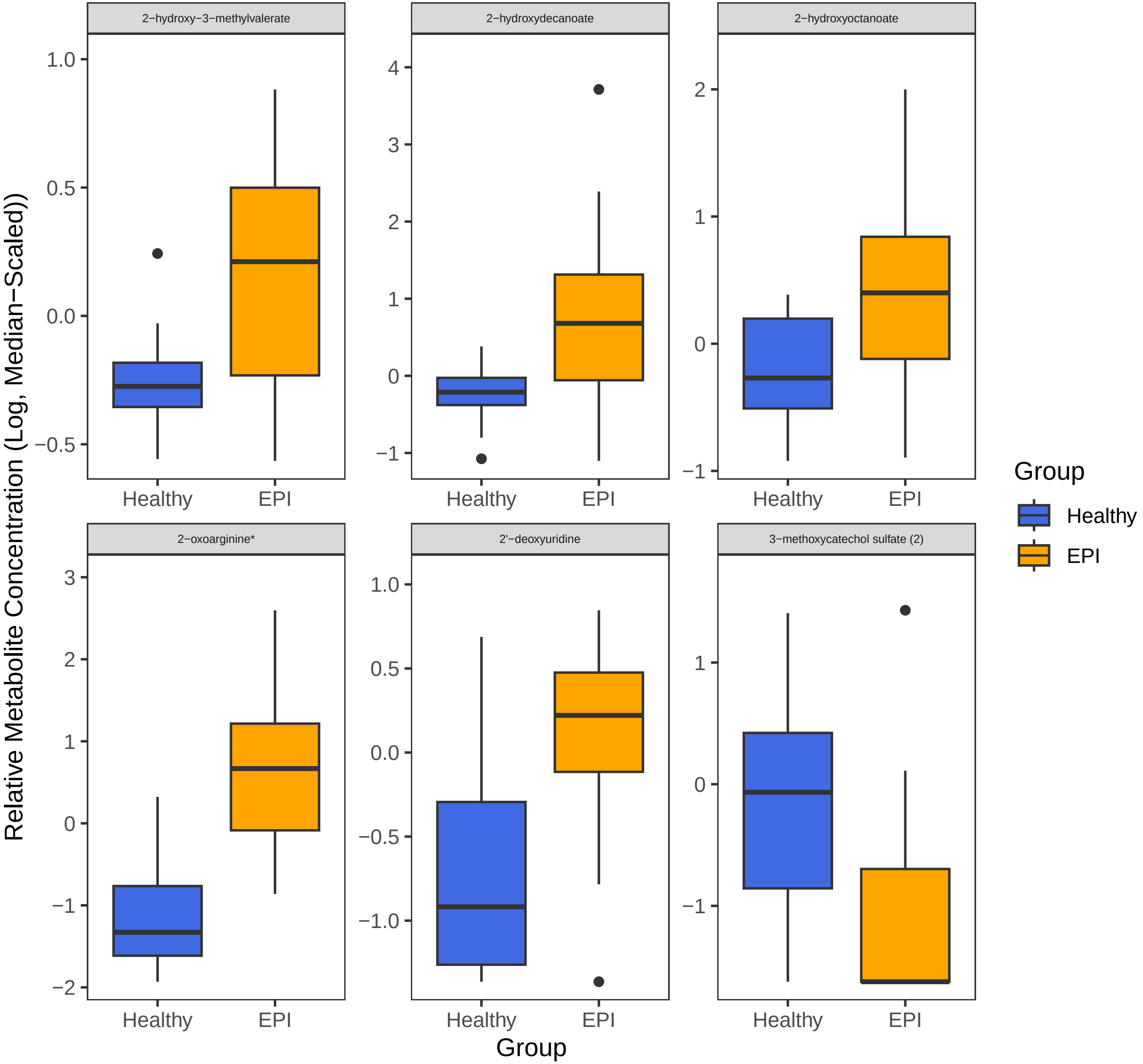
2−hydroxy−3−methylvalerate
2−hydroxydecanoate
2−hydroxyoctanoate
2−oxoarginine*
2'−deoxyuridine
3−methoxycatechol sulfate (2)
Relative Metabolite Concentration (Log, Median−Scaled))
Group
Healthy
EPI
1.0
0.5
0.0
−0.5
4
3
2
1
0
−1
−2
−1.0

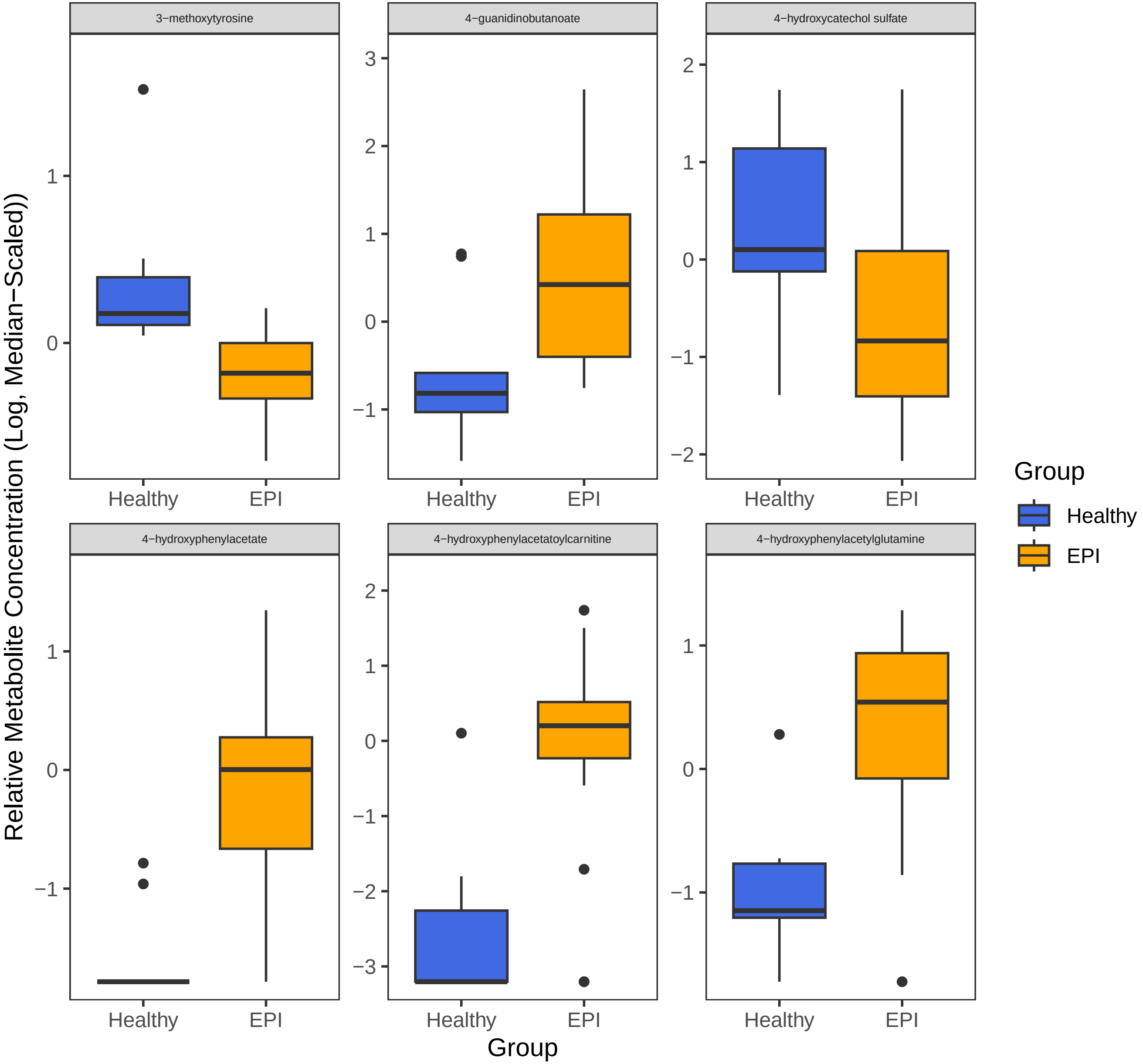

3−methoxytyrosine
4−guanidinobutanoate
4−hydroxycatechol sulfate
4−hydroxyphenylacetate
4−hydroxyphenylacetatoylcarnitine
4−hydroxyphenylacetylglutamine
Relative Metabolite Concentration (Log, Median−Scaled))
Group
Healthy
EPI

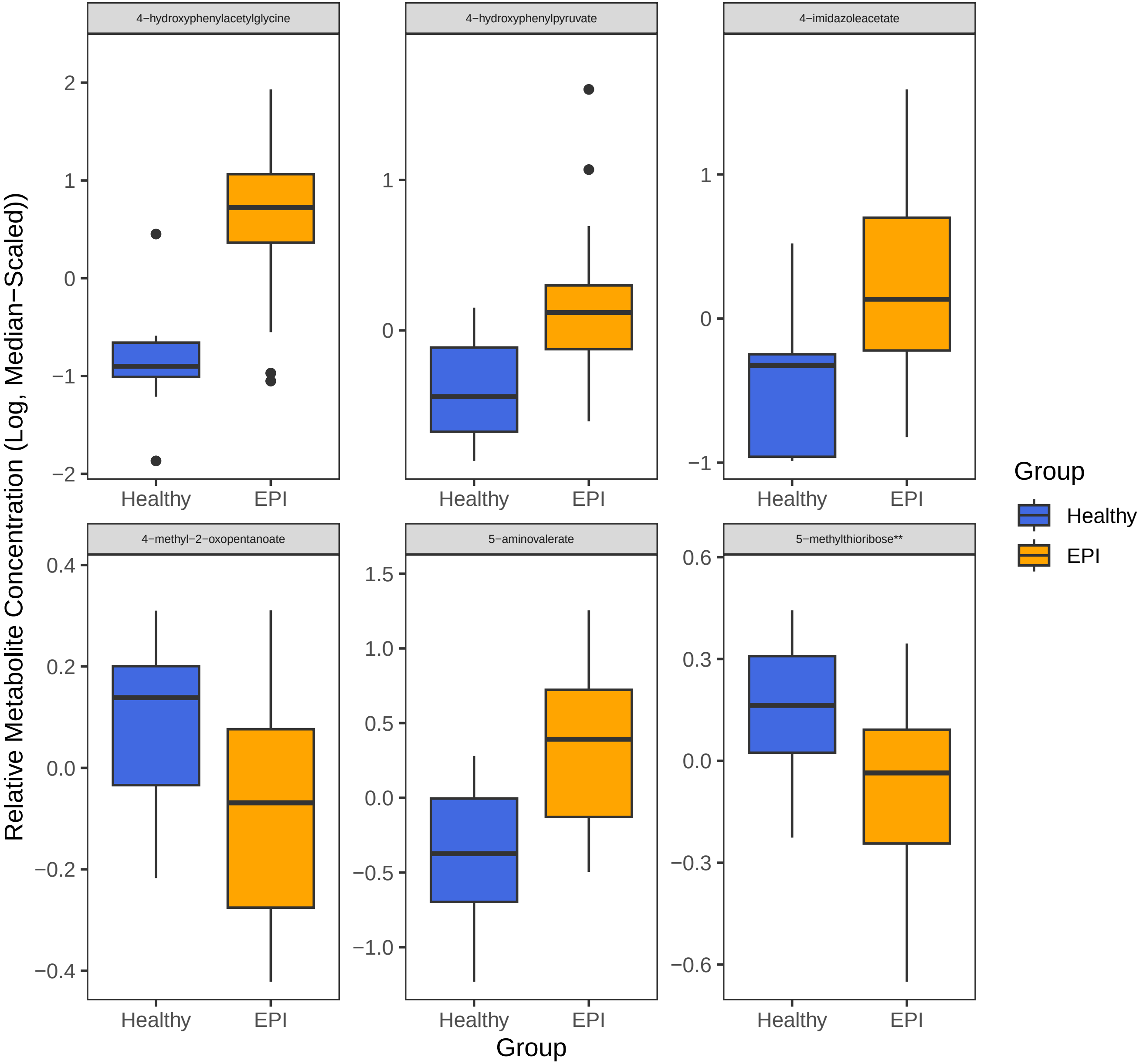
4−hydroxyphenylacetylglycine
4−hydroxyphenylpyruvate
4−imidazoleacetate
4−methyl−2−oxopentanoate
5−aminovalerate
5−methylthioribose**
Relative Metabolite Concentration (Log, Median−Scaled))
Group
Healthy
EPI
Group
Healthy
EPI
2
1
0
−1
−2
0.4
0.2
0.0
−0.2
−0.4
1.5
1.0
0.5
0.0
−0.5
−1.0
0.6
0.3
0.0
−0.3
−0.6

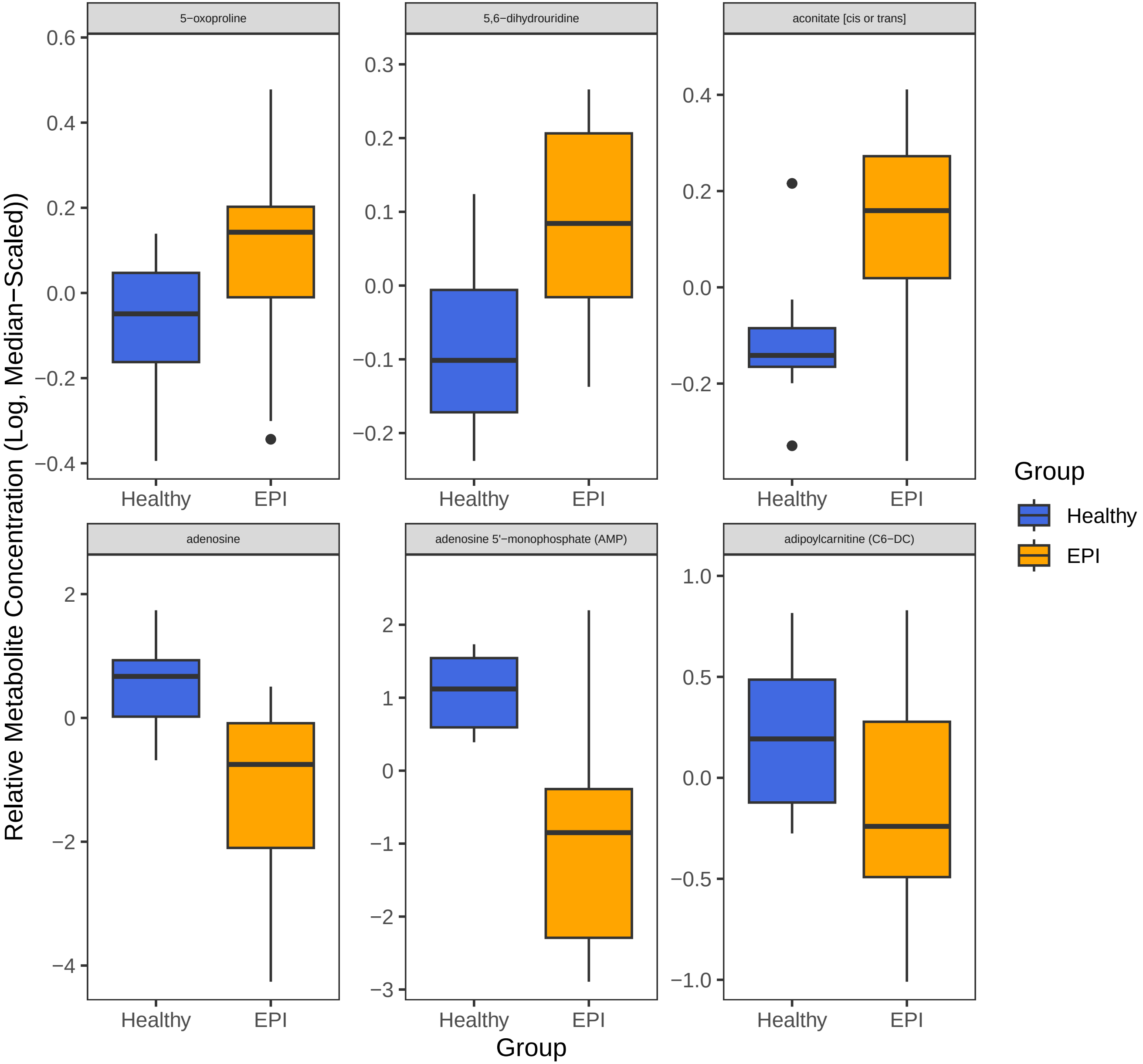
5−oxoproline
5,6−dihydrouridine
aconitate [cis or trans]
adenosine
adenosine 5'−monophosphate (AMP)
adipoylcarnitine (C6−DC)
Relative Metabolite Concentration (Log, Median−Scaled))
Group
Healthy
EPI
0.6
0.4
0.2
0.0
−0.2
−0.4
0.3
0.1
−0.1
2
1
0
−1
−2
−3
−4
1.0
0.5
−0.5
−1.0

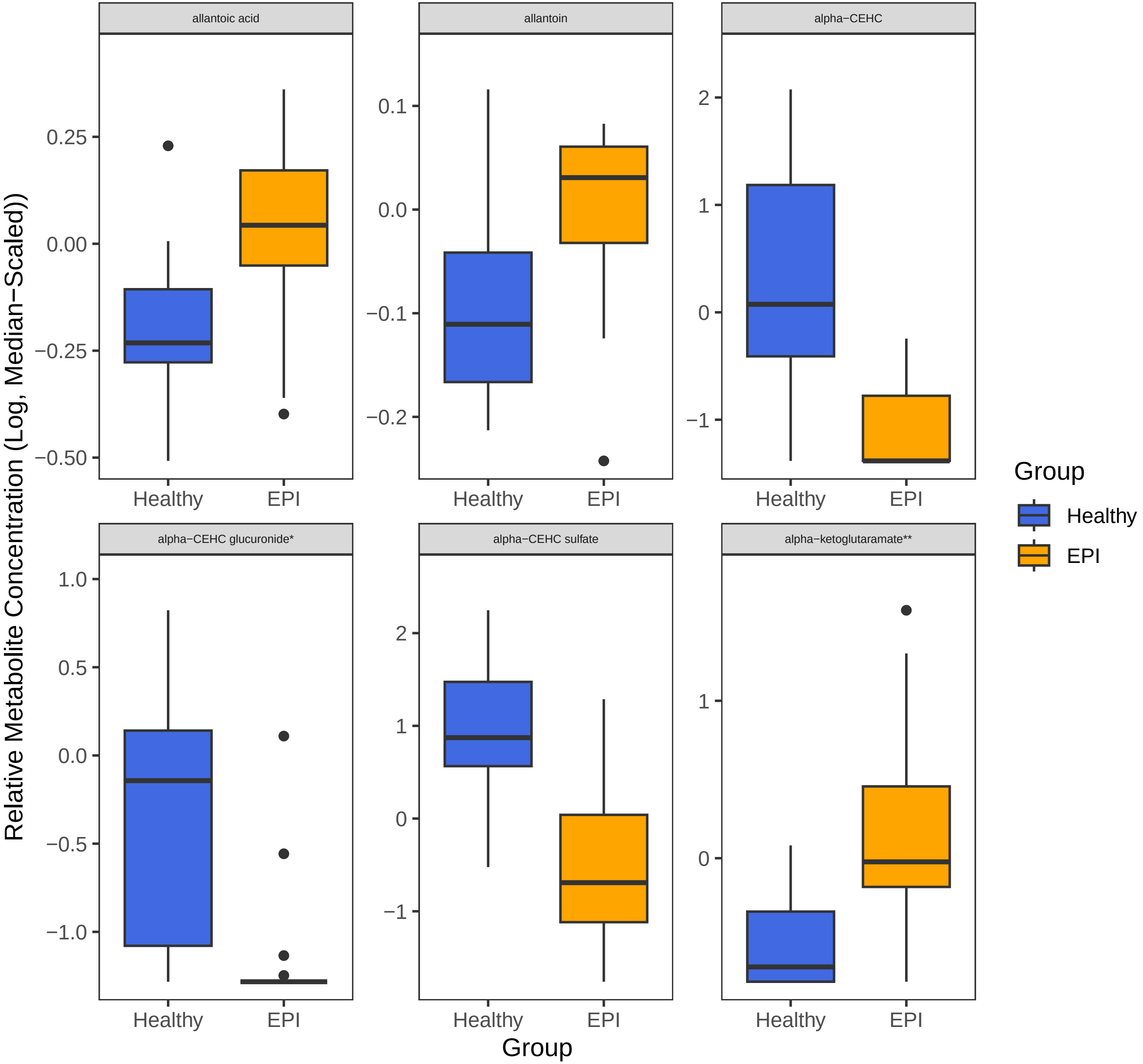
allantoic acid
allantoin
alpha−CEHC
alpha−CEHC glucuronide*
alpha−CEHC sulfate
alpha−ketoglutaramate**
0.25
0.00
−0.25
−0.50
0.1
0.0
−0.1
−0.2
2
1
0
−1
1.0
0.5
0.0
−0.5
−1.0
2
1
0
−1
1
0
Healthy
EPI
Relative Metabolite Concentration (Log, Median−Scaled))
Group
Group
Healthy
EPI

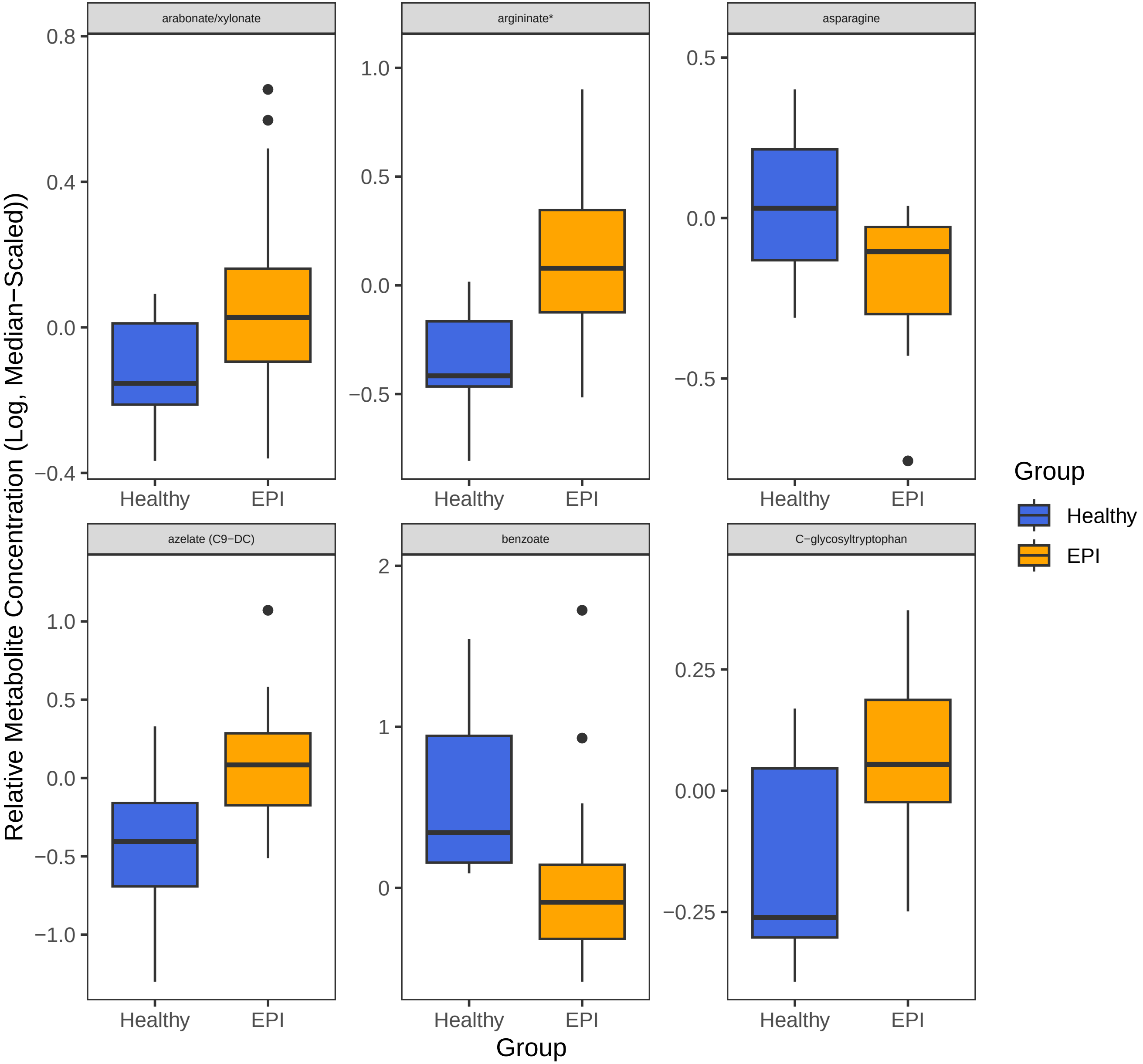
arabonate/xylonate
arginate*
asparagine
azelate (C9−DC)
benzoate
C−glycosyltryptophan
Healthy
EPI
Group
Relative Metabolite Concentration (Log, Median−Scaled))
0.8
0.4
0.0
−0.4
1.0
0.5
0.0
−0.5
1.0
0.5
0.0
−0.5
−1.0
2
1
0
0.25
0.00
−0.25

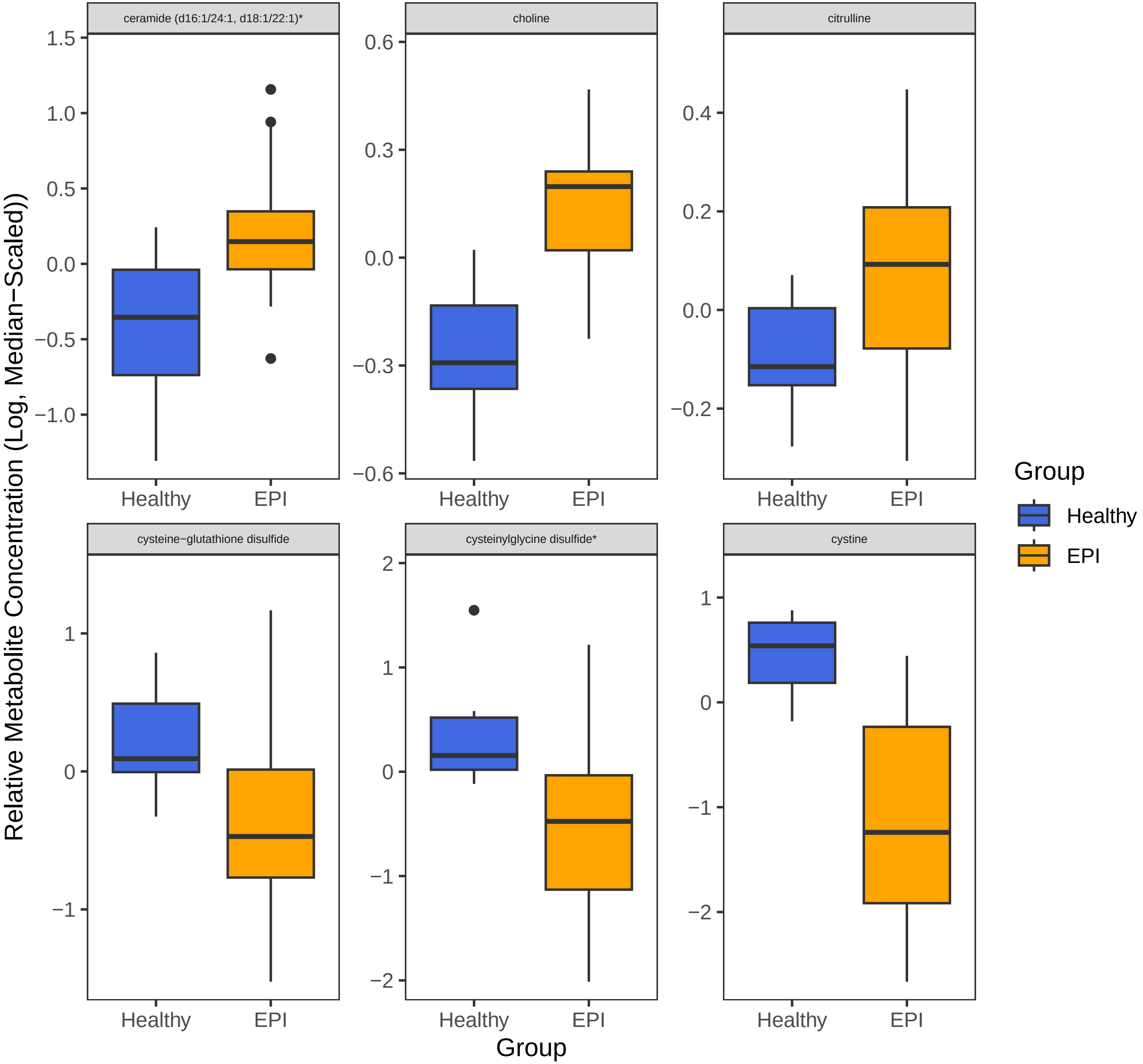
ceramide (d16:1/24:1, d18:1/22:1)*
choline
citrulline
cysteine−glutathione disulfide
cysteinylglycine disulfide*
cystine
Relative Metabolite Concentration (Log, Median−Scaled))
Group
Healthy
EPI
Group
Healthy
EPI
1.5
1.0
0.5
0.0
−0.5
−1.0
0.6
0.3
0.0
−0.3
−0.6
0.4
0.2
0.0
−0.2
1
0
−1
2
1
0
−1
−2
1
0
−1
−2

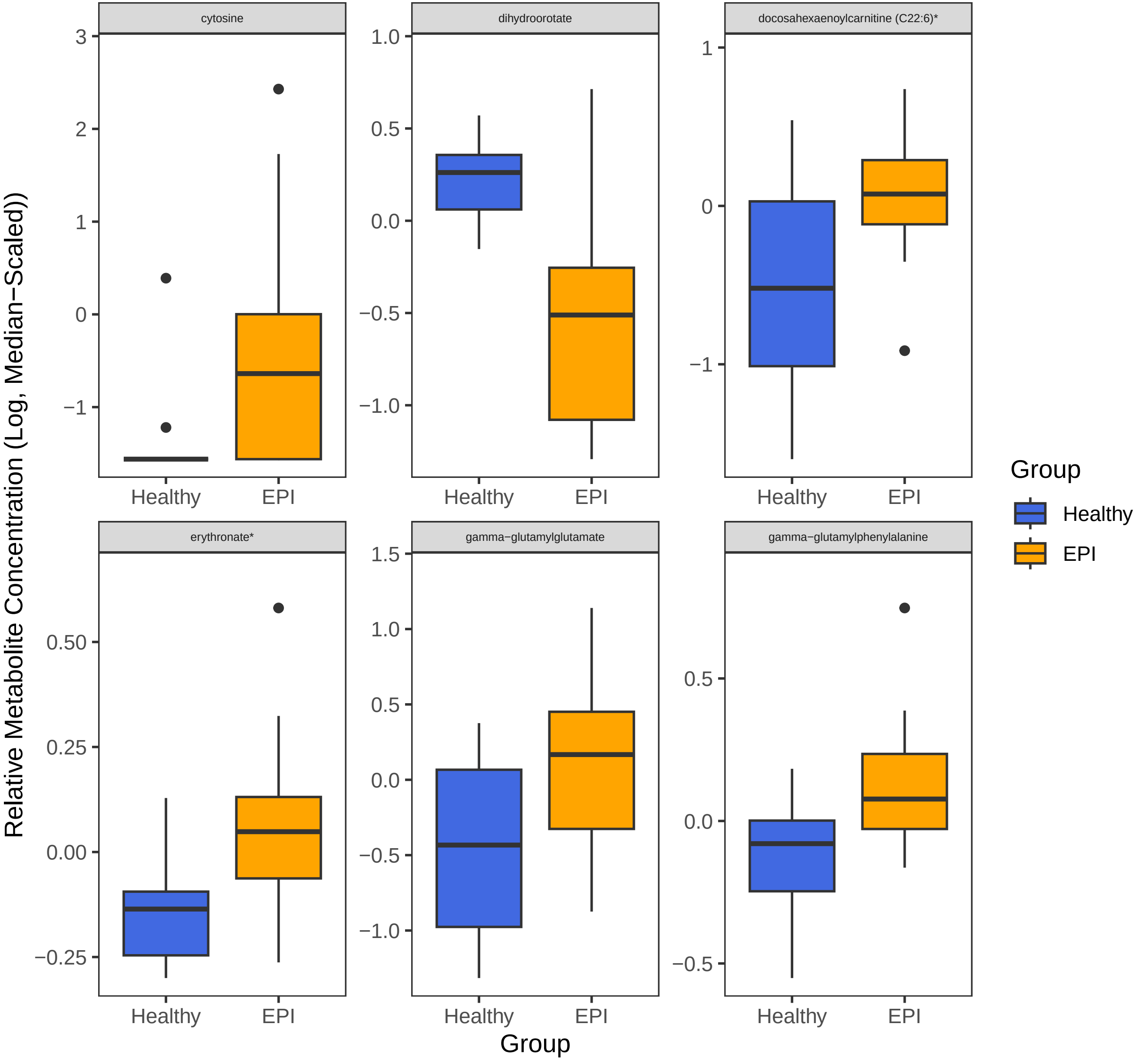
cytosine
dihydroorotate
docosahexaenoylcarnitine (C22:6)*
erythronate*
gamma−glutamylglutamate
gamma−glutamylphenylalanine
Relative Metabolite Concentration (Log, Median−Scaled))
Group
Healthy
EPI
3
2
1
0
−1
1.0
0.5
0.0
−0.5
−1.0
0.50
0.25
0.00
−0.25
1.5

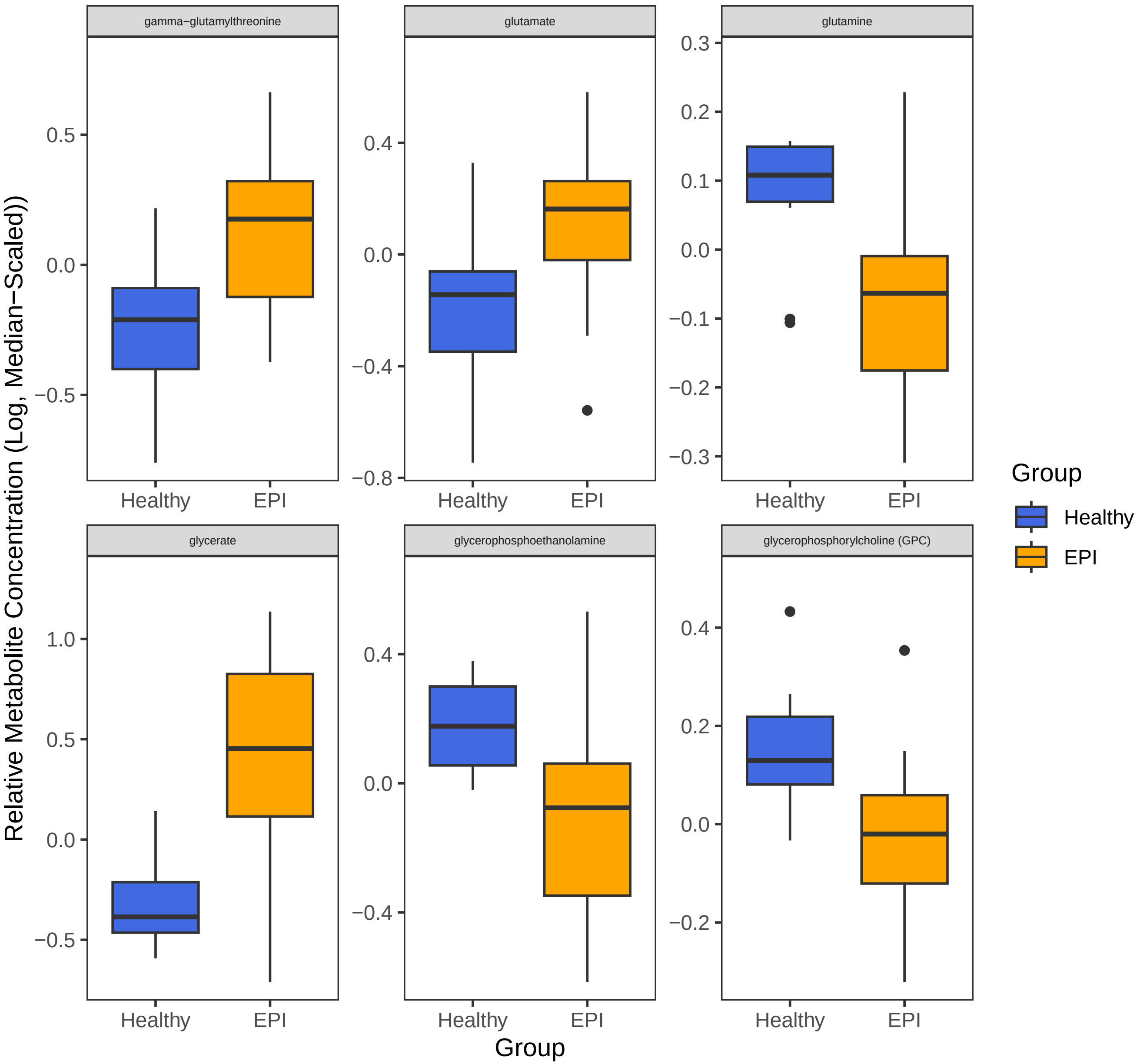
gamma−glutamylthreonine
glutamate
glutamine
glycerate
glycerophosphoethanolamine
glycerophosphorylcholine (GPC)
Relative Metabolite Concentration (Log, Median−Scaled))
Group
Healthy
EPI
0.5
0.0
−0.5
0.4
−0.4
−0.8
0.3
0.2
0.1
−0.1
−0.2
−0.3
1.0

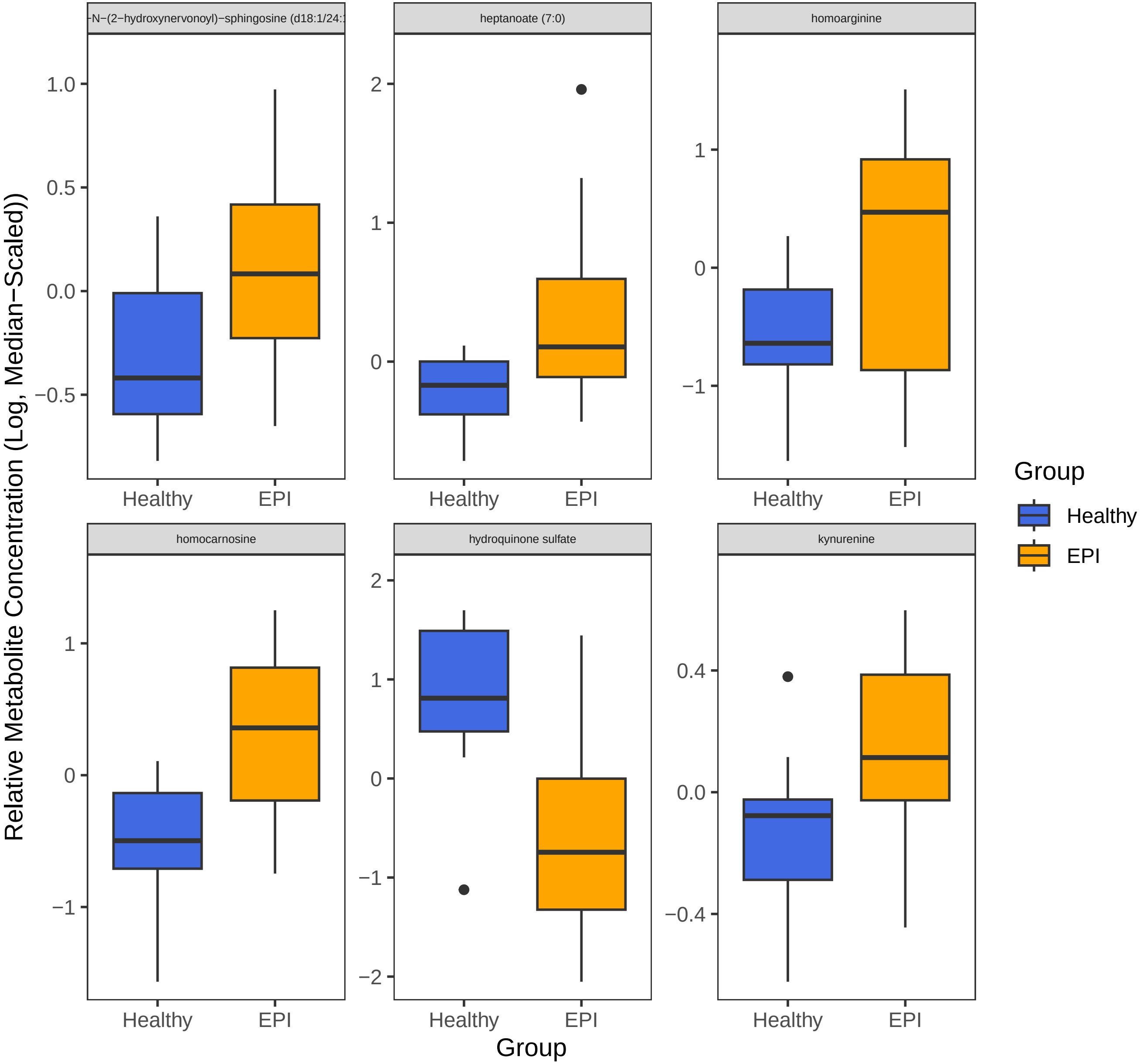
-N−(2−hydroxynervonoyl)−sphingosine (d18:1/24:1
heptanoate (7:0)
homoarginine
homocarnosine
hydroquinone sulfate
kynurenine
Relative Metabolite Concentration (Log, Median−Scaled))
Group
Healthy
EPI
Group
Healthy
EPI

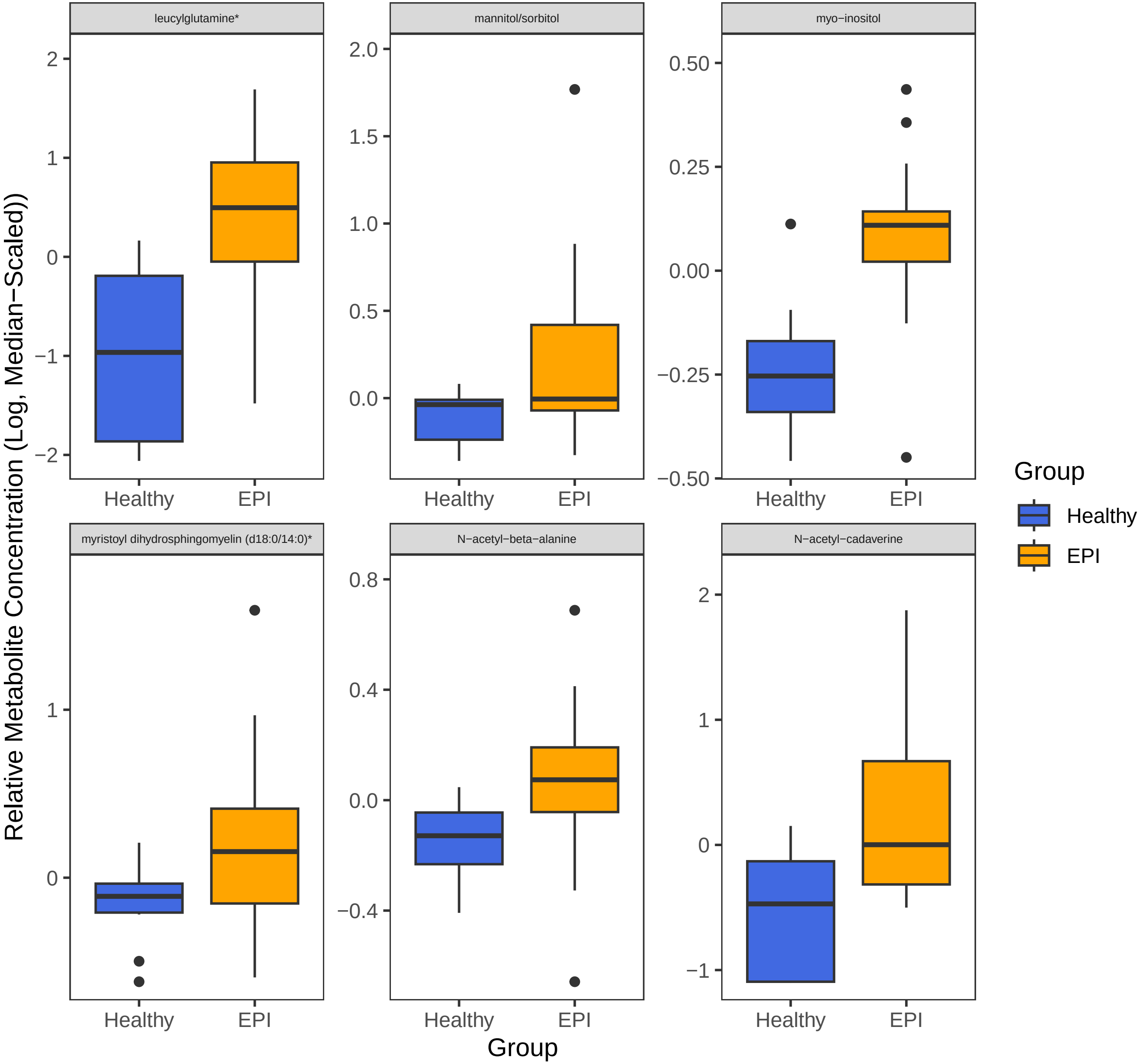
leucylglutamine*
mannitol/sorbitol
myo−inositol
myristoyl dihydrosphingomyelin (d18:0/14:0)*
N−acetyl−beta−alanine
N−acetyl−cadaverine
Relative Metabolite Concentration (Log, Median−Scaled))
Group
Healthy
EPI
Group
Healthy
EPI

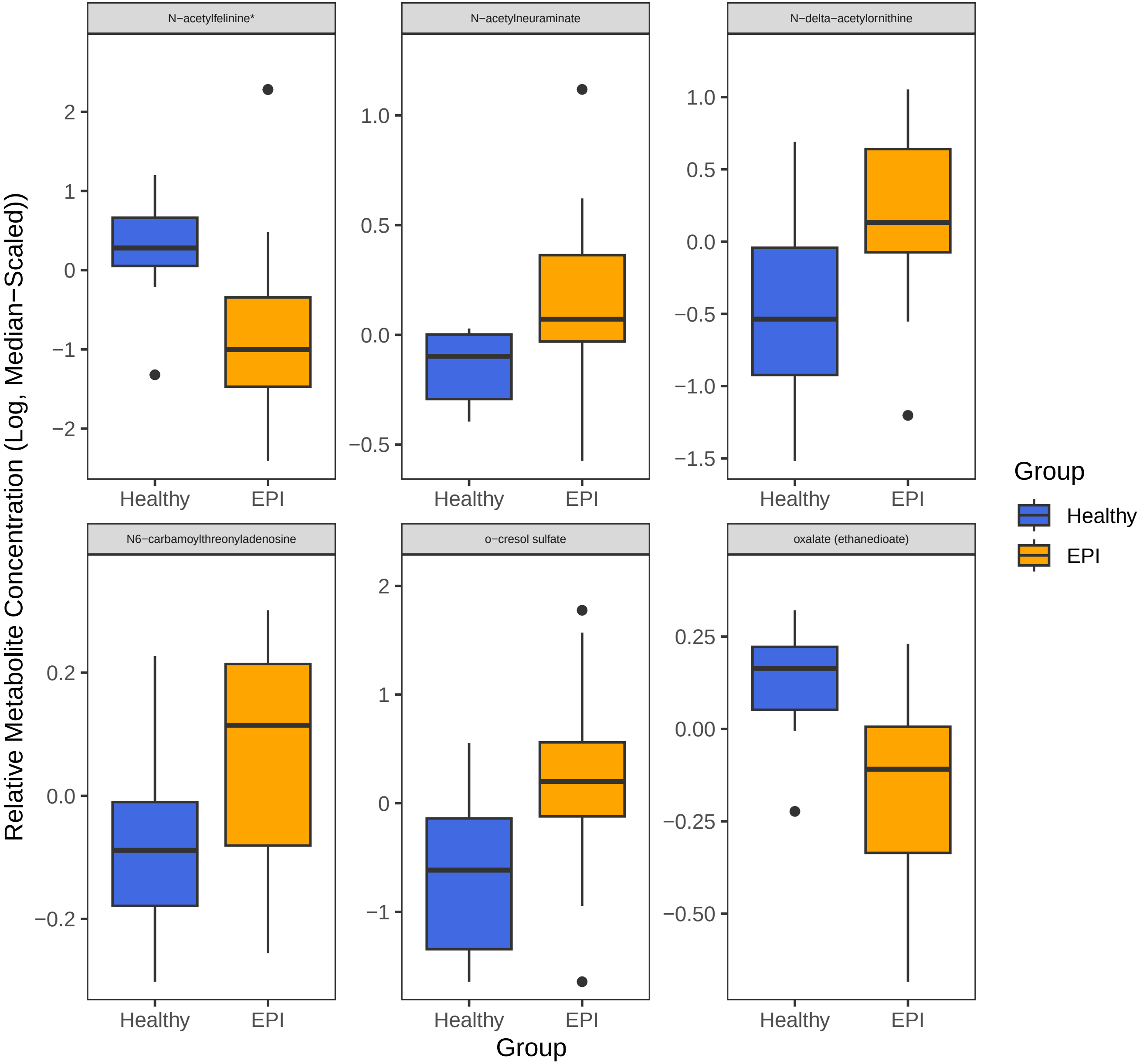
N−acetylfelinine*
N−acetylneuraminate
N−delta−acetylornithine
N6−carbamoylthreonyladenosine
o−cresol sulfate
oxalate (ethanedioate)
Relative Metabolite Concentration (Log, Median−Scaled))
Group
Healthy
EPI
Group
Healthy
EPI

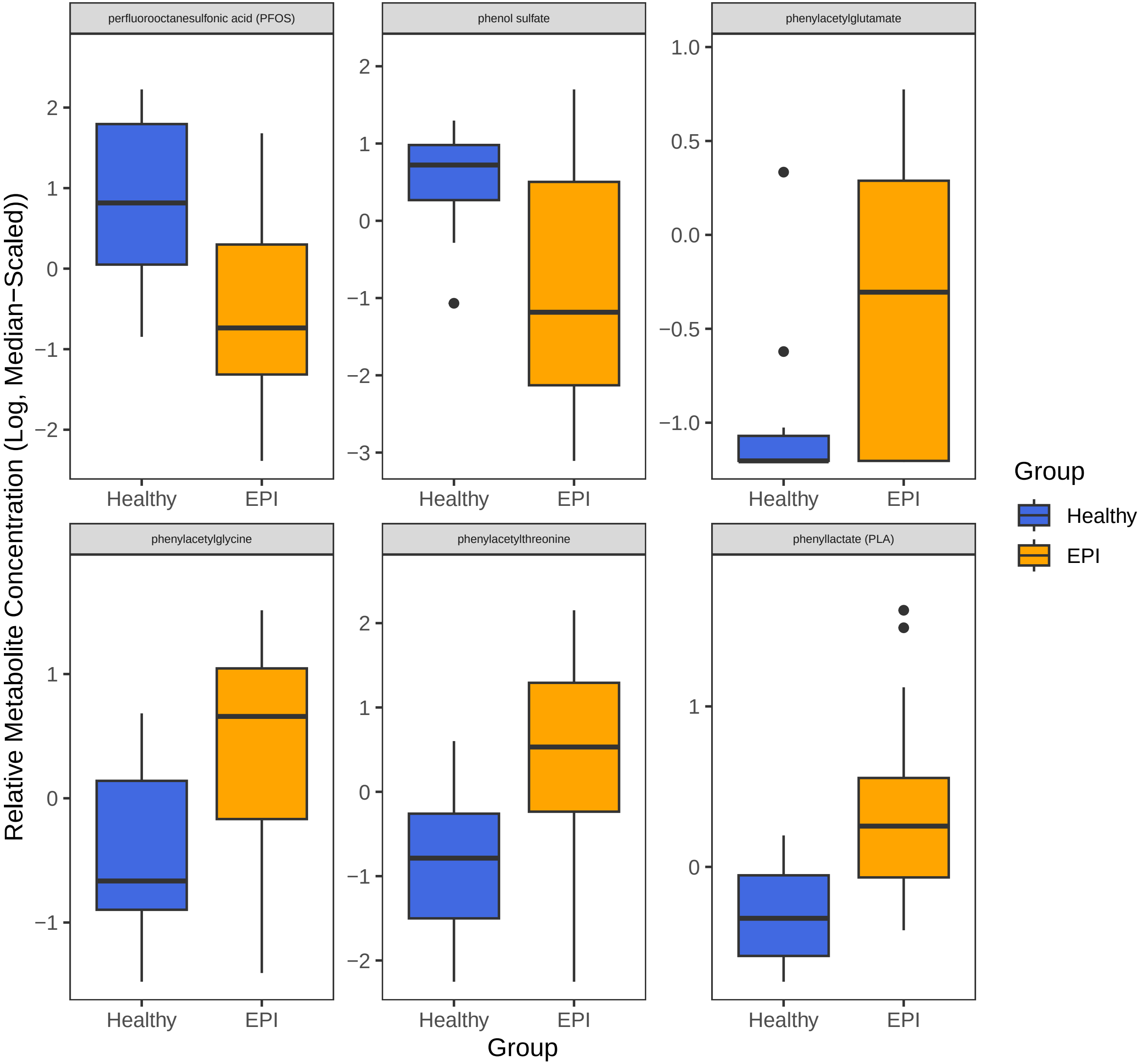
perfluorooctanesulfonic acid (PFOS)
phenol sulfate
phenylacetylglutamate
phenylacetylglycine
phenylacetylthreonine
phenyllactate (PLA)
Relative Metabolite Concentration (Log, Median−Scaled))
Group
Healthy
EPI
Group
Healthy
EPI

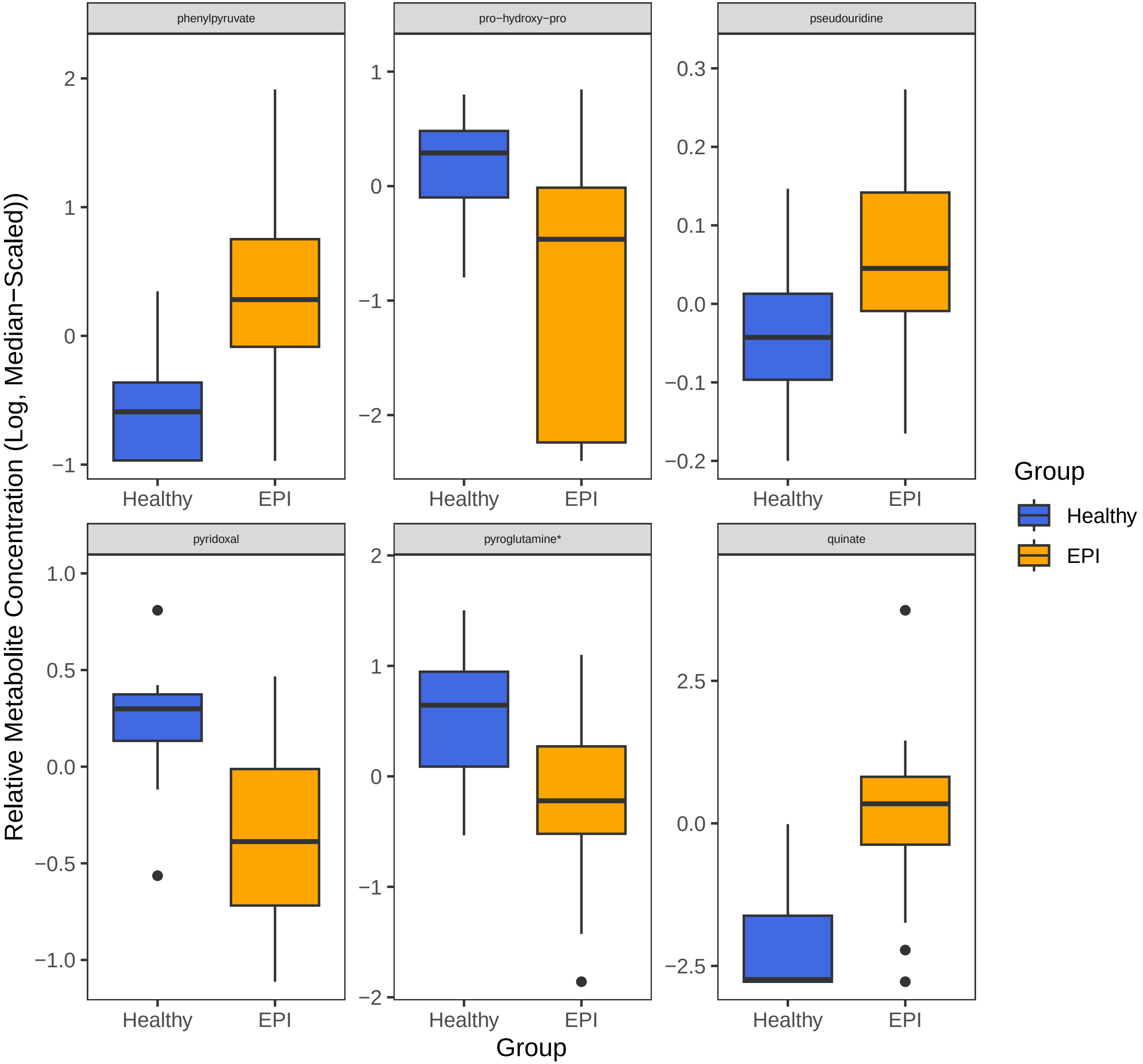
phenylpyruvate
pro−hydroxy−pro
pseudouridine
pyridoxal
pyroglutamine*
quinate
Relative Metabolite Concentration (Log, Median−Scaled))
Group
Healthy
EPI
2
1
0
−1
−2
0.3
0.2
0.1
0.0
−0.1
−0.2
1.0
0.5
−0.5
−1.0
2.5
−2.5

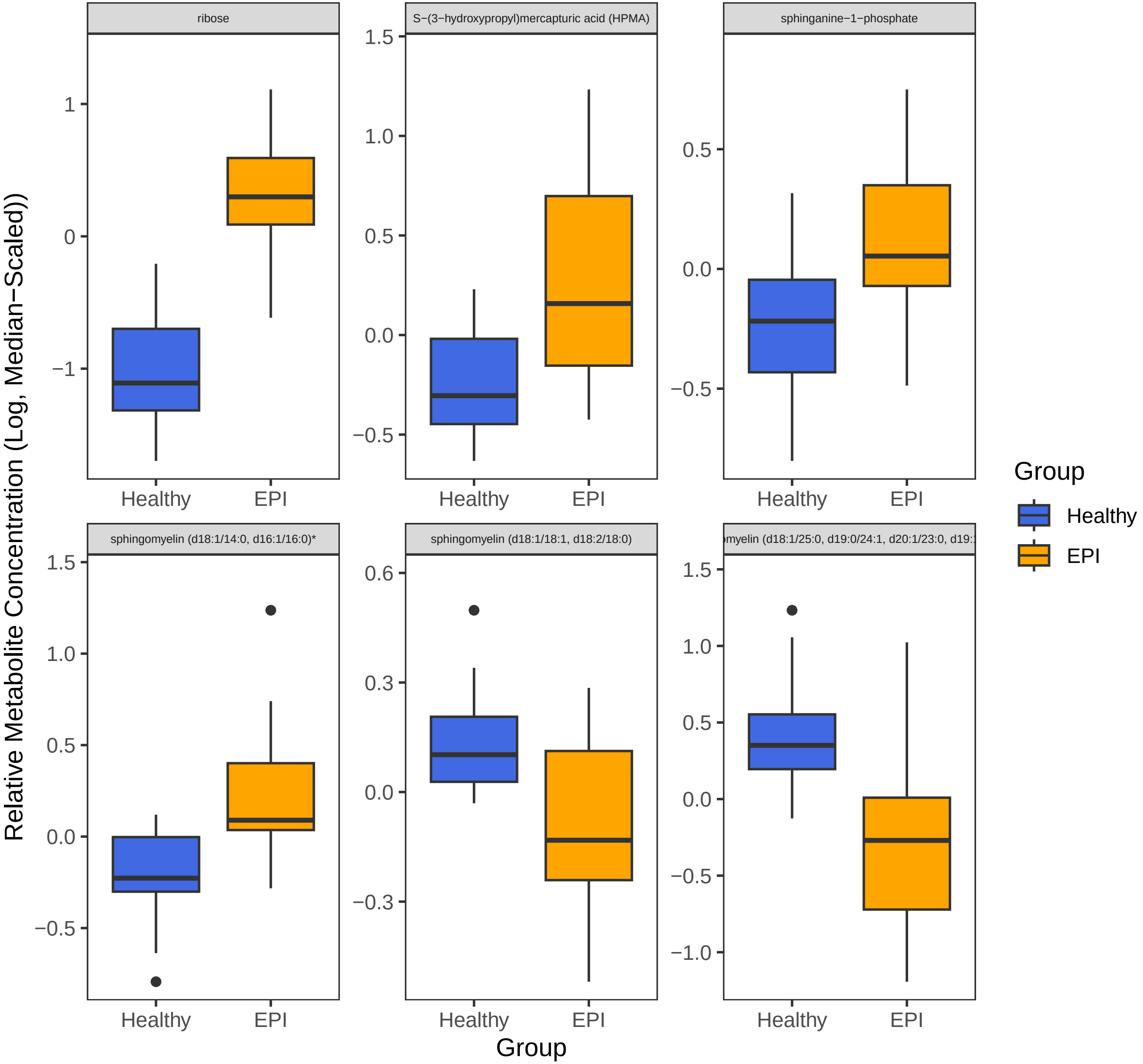
ribose
S−(3−hydroxypropyl)mercapturic acid (HPMA)
sphinganine−1−phosphate
sphingomyelin (d18:1/14:0, d16:1/16:0)*
sphingomyelin (d18:1/18:1, d18:2/18:0)
myelin (d18:1/25:0, d19:0/24:1, d20:1/23:0, d19:
Relative Metabolite Concentration (Log, Median−Scaled))
Healthy
EPI
Group
Group
Healthy
EPI

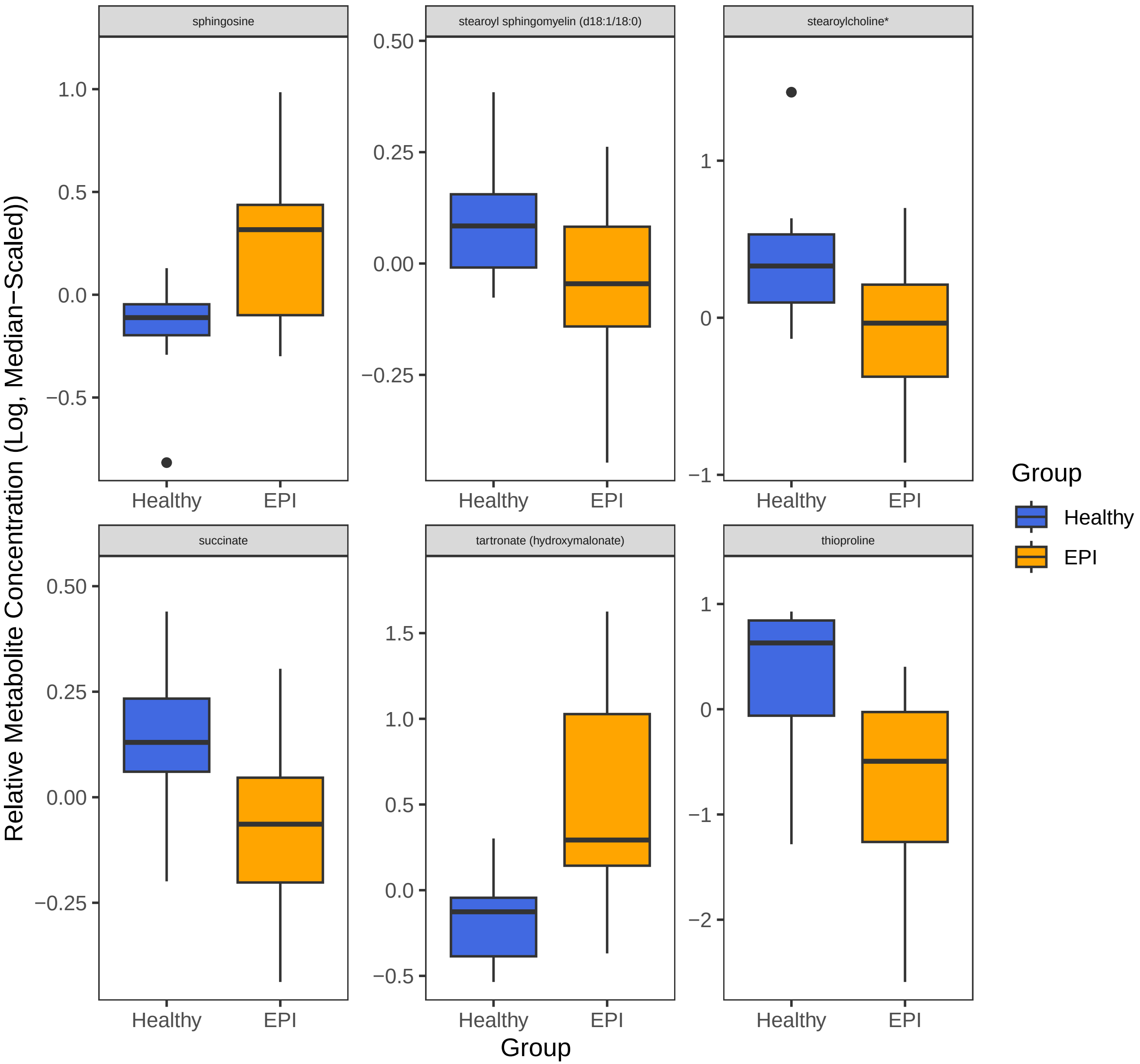
sphingosine
stearoyl sphingomyelin (d18:1/18:0)
stearoylcholine*
succinate
tartronate (hydroxymalonate)
thioproline
Relative Metabolite Concentration (Log, Median−Scaled))
Healthy
EPI
Group
Group
Healthy
EPI
1.0
0.5
0.0
−0.5
0.50
0.25
0.00
−0.25
1
0
−1
1.5
−2

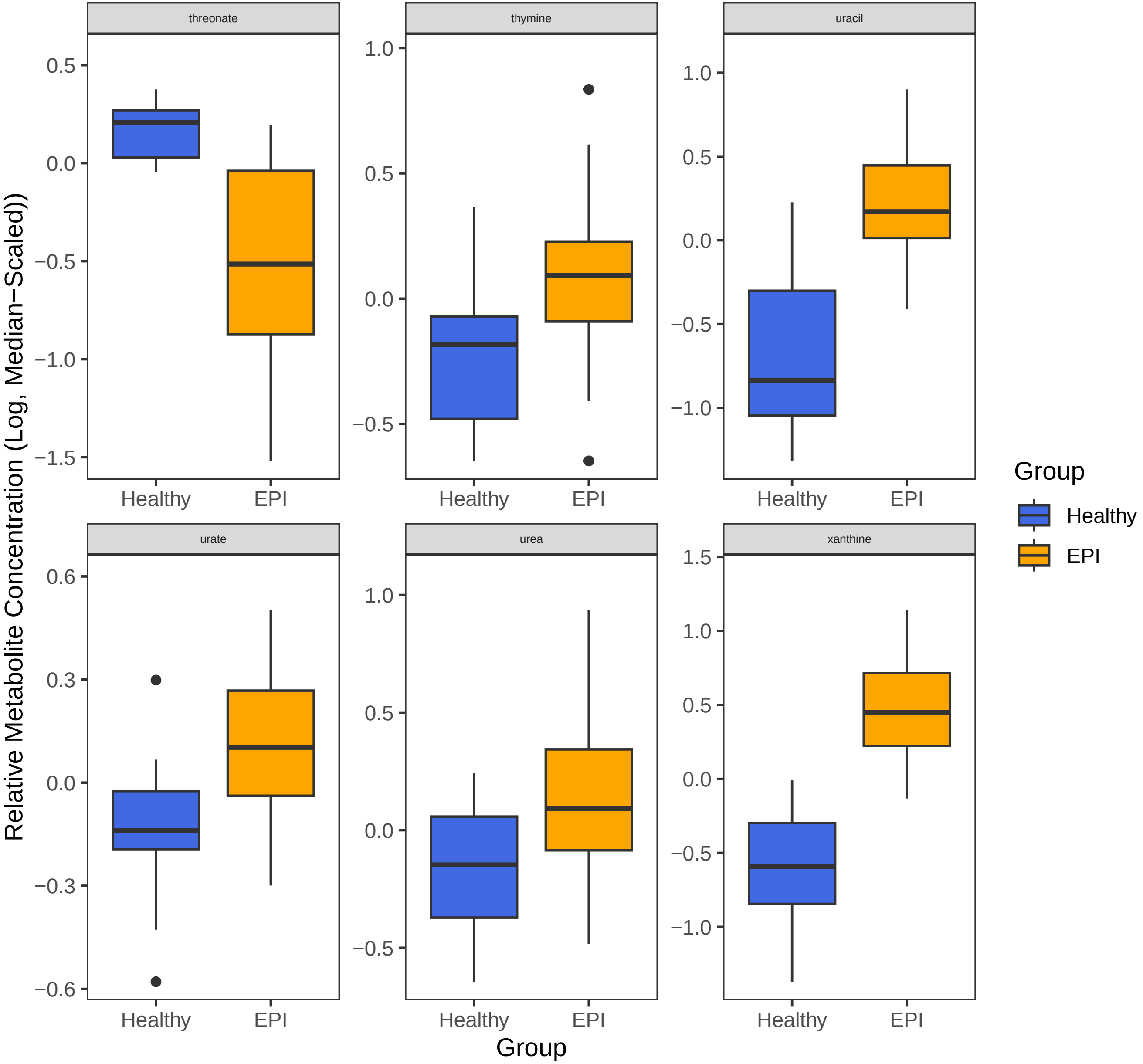
threonate
thymine
uracil
urate
urea
xanthine
Healthy
EPI
Relative Metabolite Concentration (Log, Median−Scaled))
Group
0.5
0.0
−0.5
−1.0
−1.5
1.0
0.6
0.3
−0.3
−0.6
1.5
