# Supplementary material for: Untargeted Analysis of Serum Metabolomes in Dogs with Exocrine Pancreatic Insufficiency
Source: Animals (Basel). 2023 Jul 14;13(14):2313. doi: 10.3390/ani13142313 (PMC10376357; doi:10.3390/ani13142313)
Supplement: Supplementary file 1 [file animals-13-02313-s001.zip › animals-2380457-supplementary/Supplemental Info/File S5.pdf]

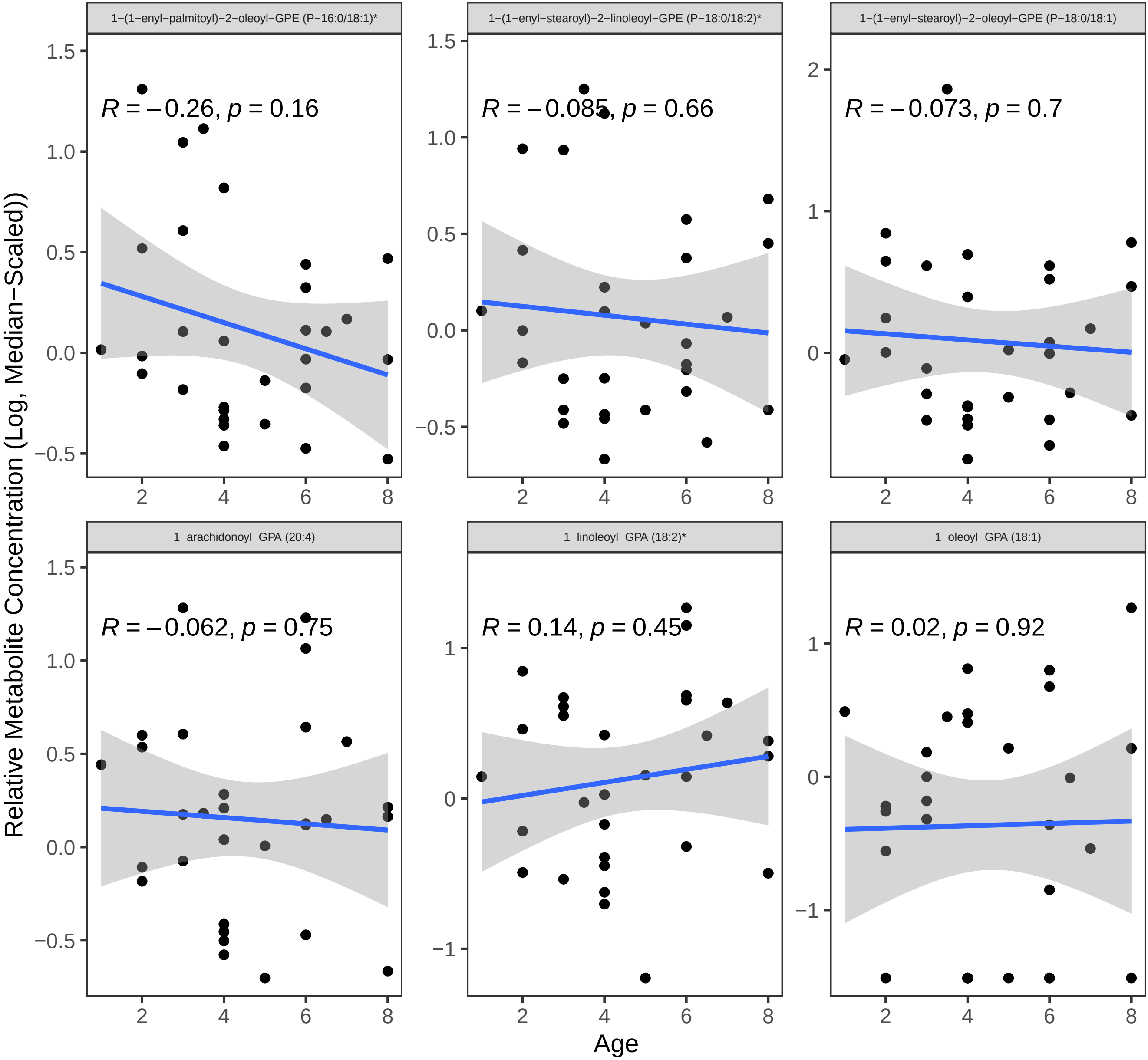
1−(1−enyl−palmitoyl)−2−oleoyl−GPE (P−16:0/18:1)*
R = −0.26, p = 0.16
1−(1−enyl−stearoyl)−2−linoleoyl−GPE (P−18:0/18:2)*
R = −0.085, p = 0.66
1−(1−enyl−stearoyl)−2−oleoyl−GPE (P−18:0/18:1)
R = −0.073, p = 0.7
1−arachidonoyl−GPA (20:4)
R = −0.062, p = 0.75
1−linoleoyl−GPA (18:2)*
R = 0.14, p = 0.45
1−oleoyl−GPA (18:1)
R = 0.02, p = 0.92
Relative Metabolite Concentration (Log, Median−Scaled))
Age

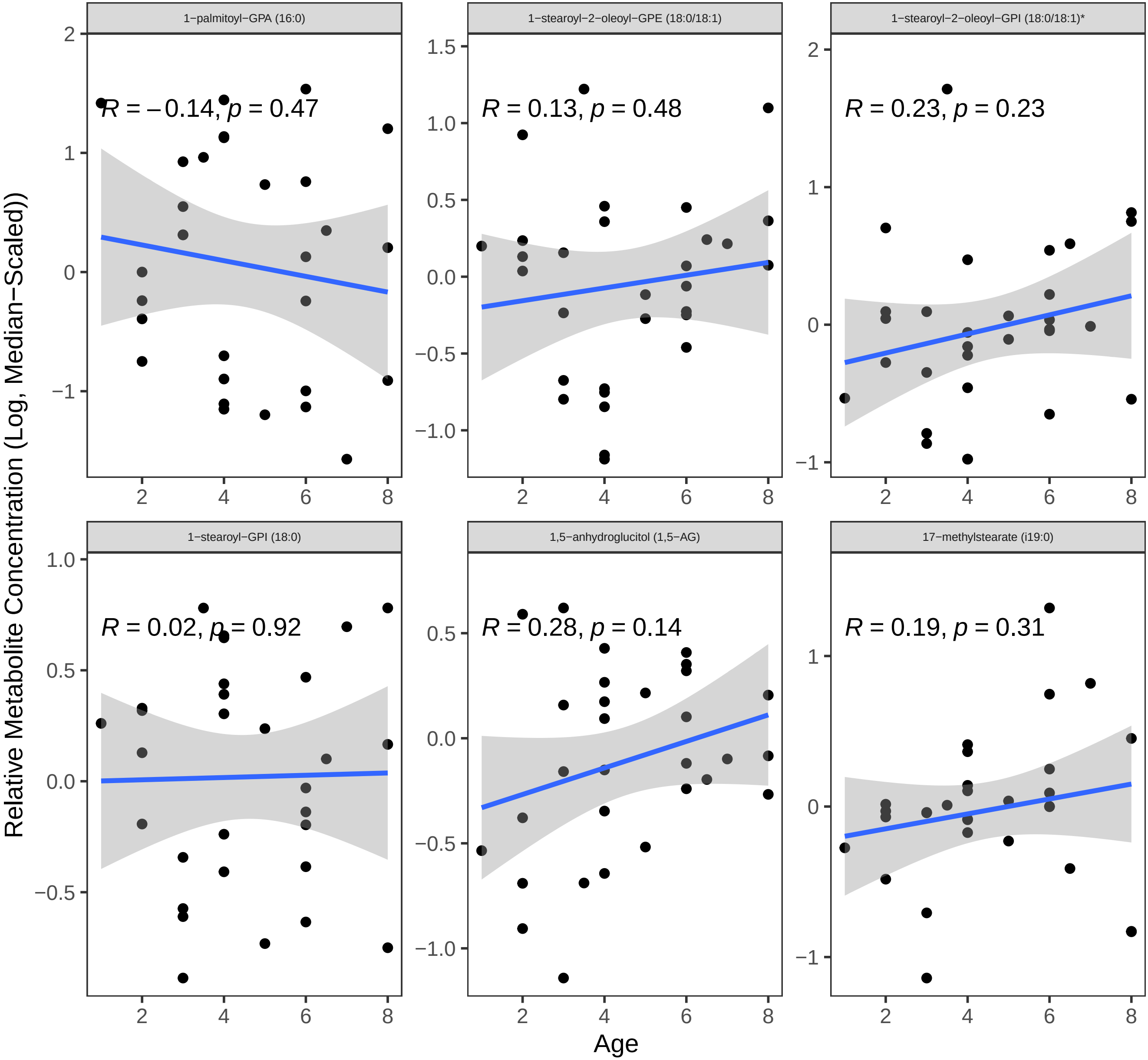
1−palmitoyl−GPA (16:0)
R = −0.14, p = 0.47
1−stearoyl−2−oleoyl−GPE (18:0/18:1)
R = 0.13, p = 0.48
1−stearoyl−2−oleoyl−GPI (18:0/18:1)*
R = 0.23, p = 0.23
1−stearoyl−GPI (18:0)
R = 0.02, p = 0.92
1,5−anhydroglucitol (1,5−AG)
R = 0.28, p = 0.14
17−methylstearate (i19:0)
R = 0.19, p = 0.31
Relative Metabolite Concentration (Log, Median−Scaled))
Age

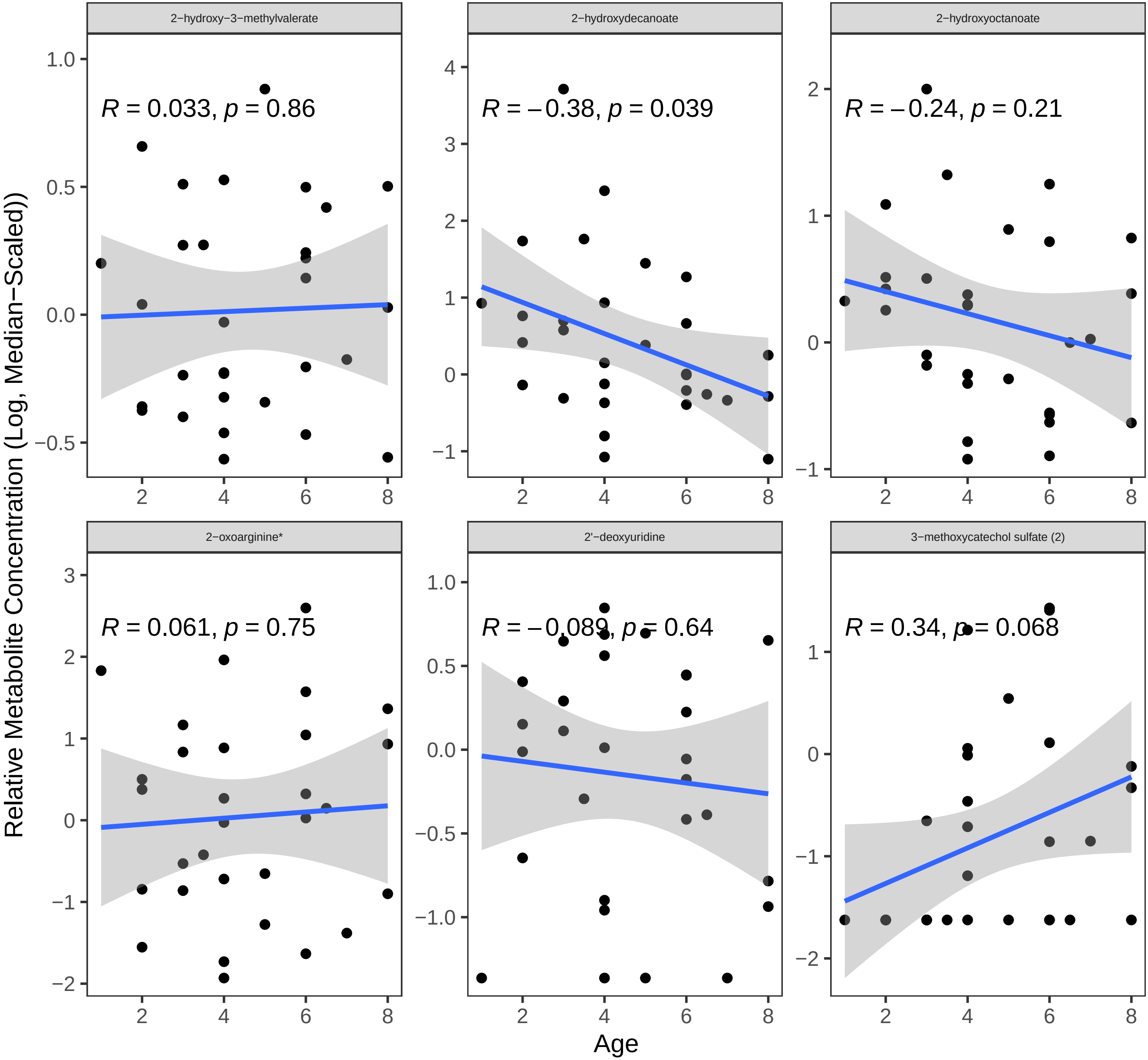
2−hydroxy−3−methylvalerate
R = 0.033, p = 0.86
2−hydroxydecanoate
R = −0.38, p = 0.039
2−hydroxyoctanoate
R = −0.24, p = 0.21
2−oxoarginine*
R = 0.061, p = 0.75
2'−deoxyuridine
R = −0.089, p = 0.64
3−methoxycatechol sulfate (2)
R = 0.34, p = 0.068
Relative Metabolite Concentration (Log, Median−Scaled))
Age

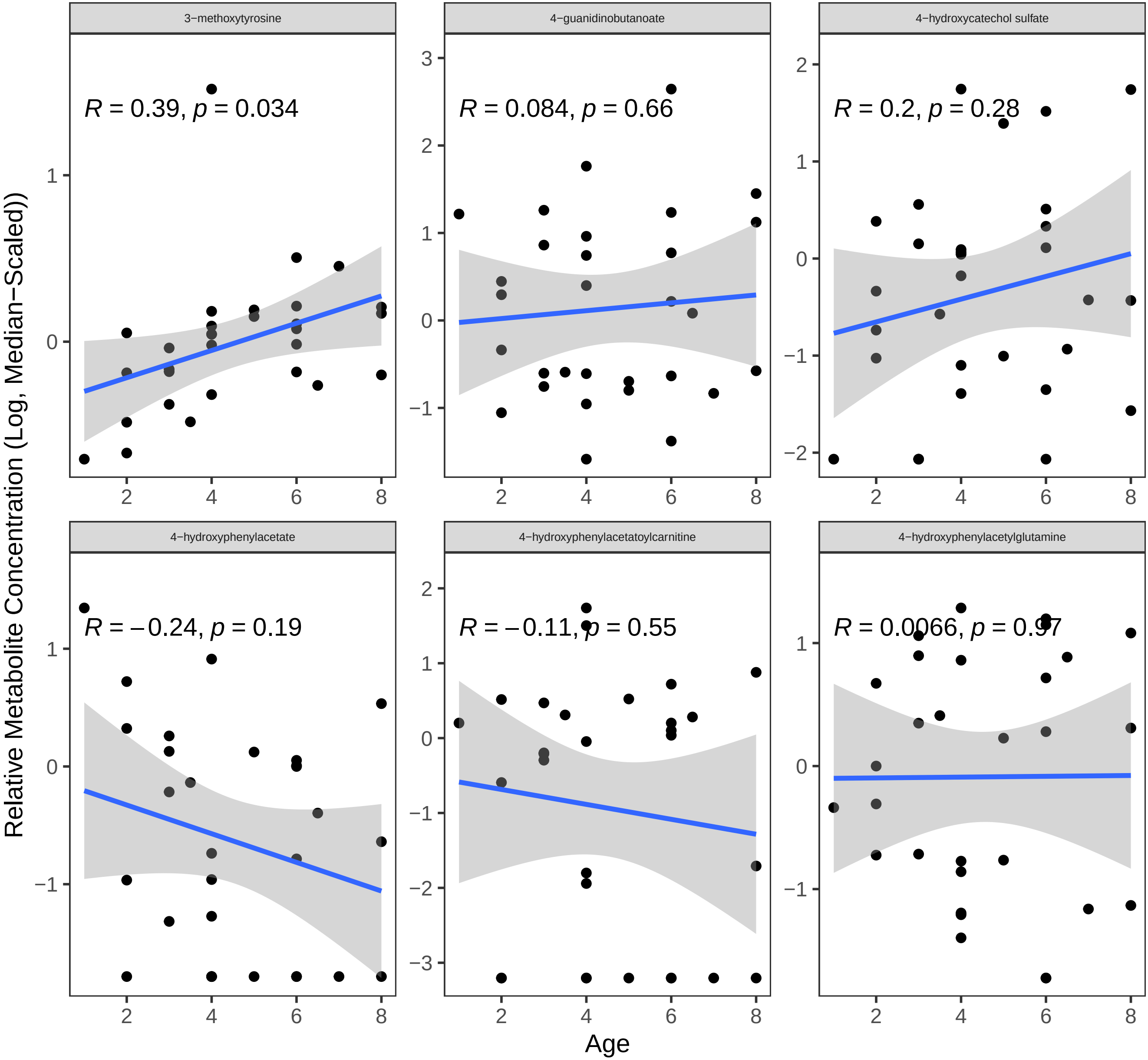
3−methoxytyrosine
R = 0.39, p = 0.034
4−guanidinobutanoate
R = 0.084, p = 0.66
4−hydroxycatechol sulfate
R = 0.2, p = 0.28
4−hydroxyphenylacetate
R = −0.24, p = 0.19
4−hydroxyphenylacetatoylcarnitine
R = −0.11, p = 0.55
4−hydroxyphenylacetylglutamine
R = 0.0066, p = 0.97
Relative Metabolite Concentration (Log, Median−Scaled))
Age

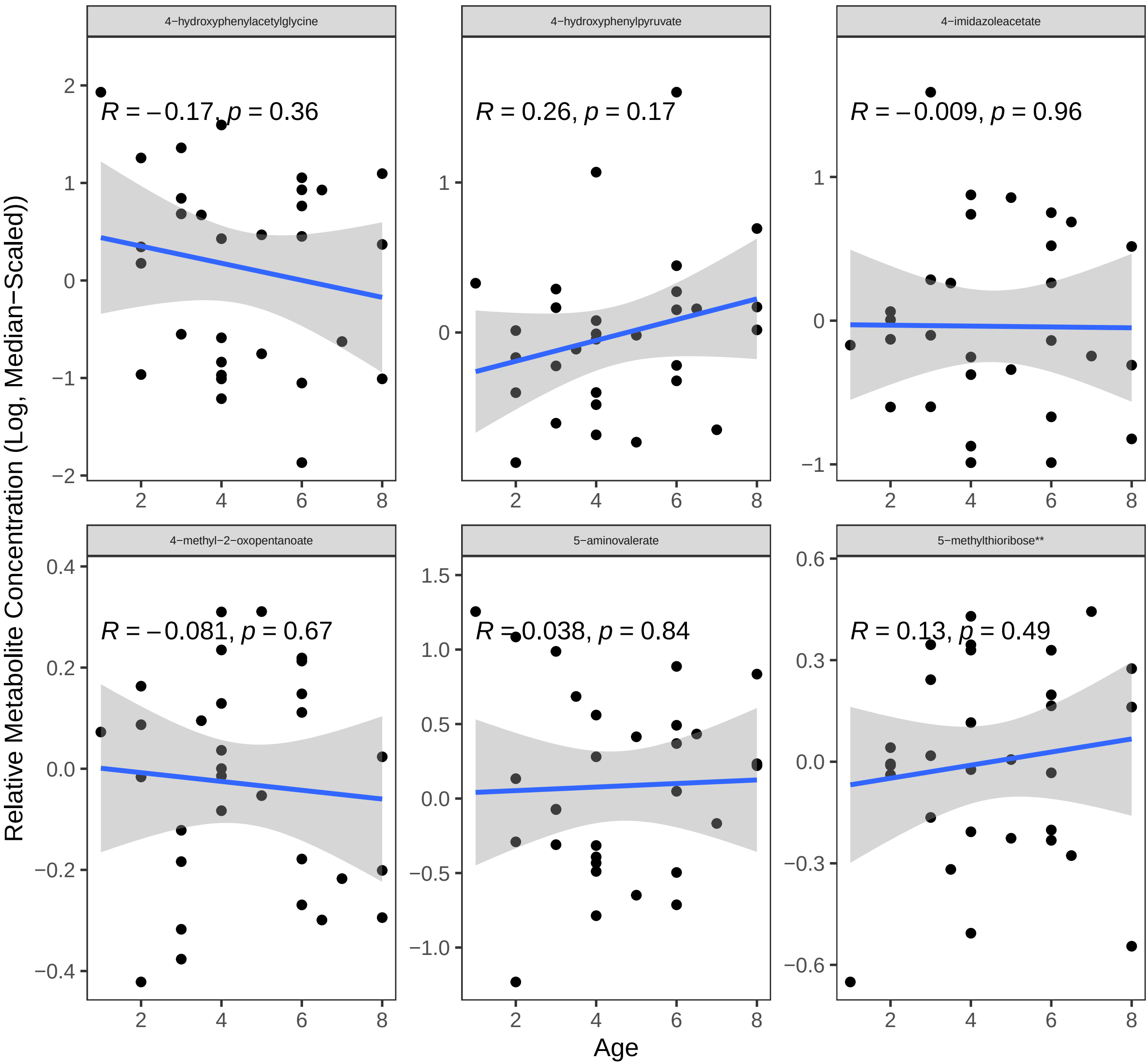
4−hydroxyphenylacetylglycine
R = −0.17, p = 0.36
4−hydroxyphenylpyruvate
R = 0.26, p = 0.17
4−imidazoleacetate
R = −0.009, p = 0.96
4−methyl−2−oxopentanoate
R = −0.081, p = 0.67
5−aminovalerate
R = 0.038, p = 0.84
5−methylthioribose**
R = 0.13, p = 0.49
Relative Metabolite Concentration (Log, Median−Scaled))
Age

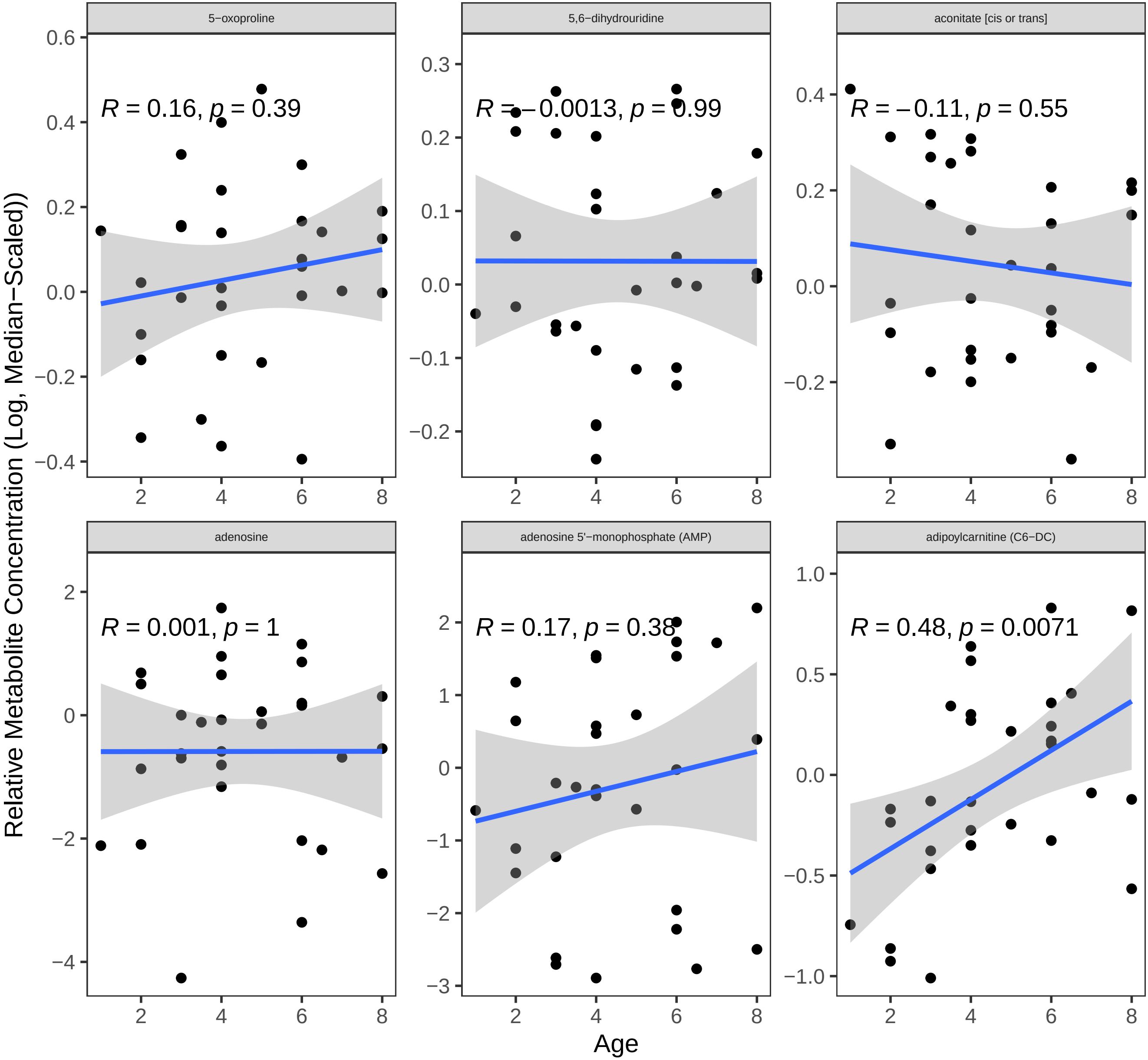
5−oxoproline
R = 0.16, p = 0.39
5,6−dihydrouridine
R = −0.0013, p = 0.99
aconitate [cis or trans]
R = −0.11, p = 0.55
adenosine
R = 0.001, p = 1
adenosine 5'−monophosphate (AMP)
R = 0.17, p = 0.38
adipoylcarnitine (C6−DC)
R = 0.48, p = 0.0071
Relative Metabolite Concentration (Log, Median−Scaled))
Age

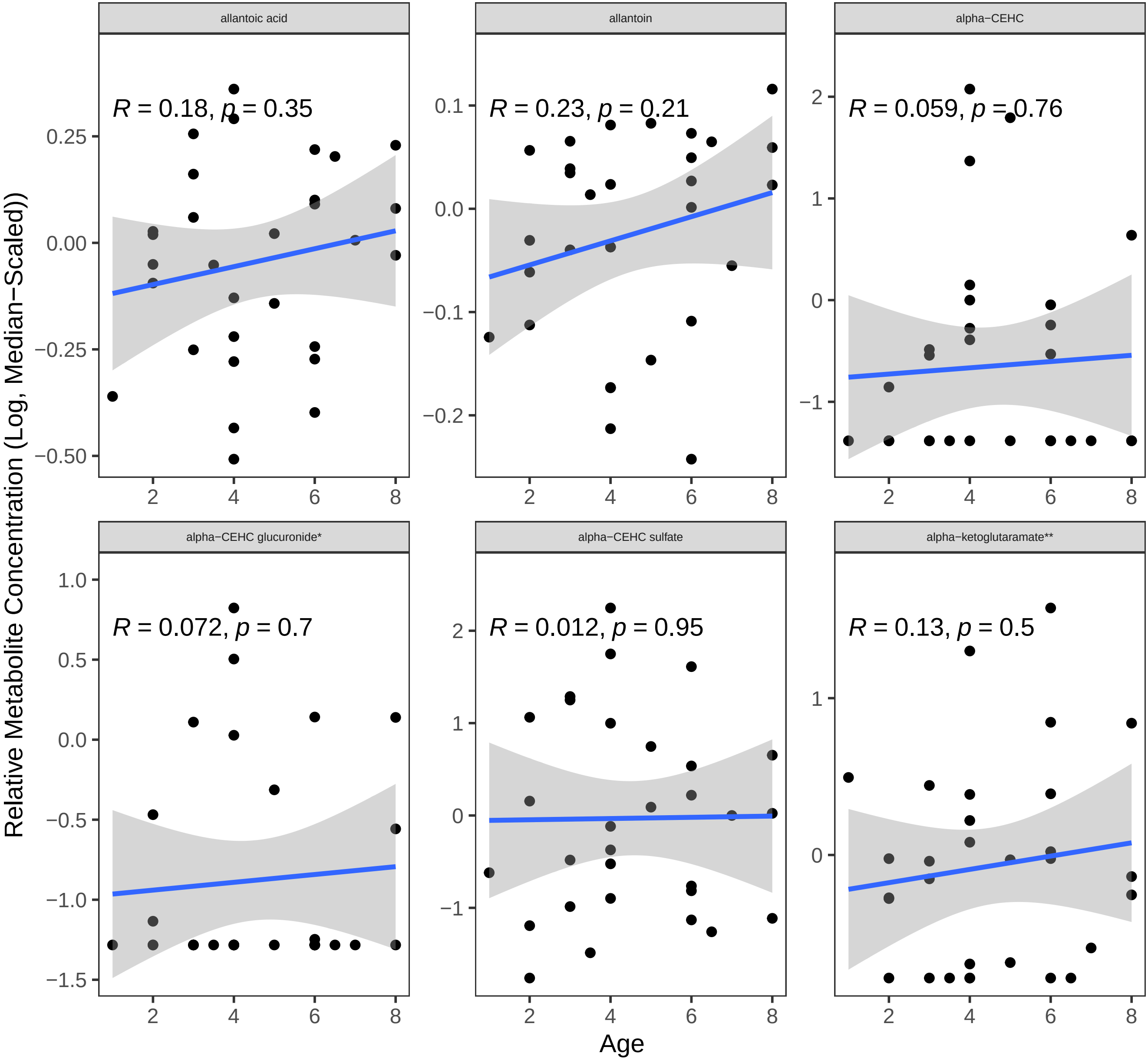
allantoic acid
R = 0.18, p = 0.35
allantoin
R = 0.23, p = 0.21
alpha−CEHC
R = 0.059, p = 0.76
alpha−CEHC glucuronide*
R = 0.072, p = 0.7
alpha−CEHC sulfate
R = 0.012, p = 0.95
alpha−ketoglutaramate**
R = 0.13, p = 0.5
Relative Metabolite Concentration (Log, Median−Scaled))
Age

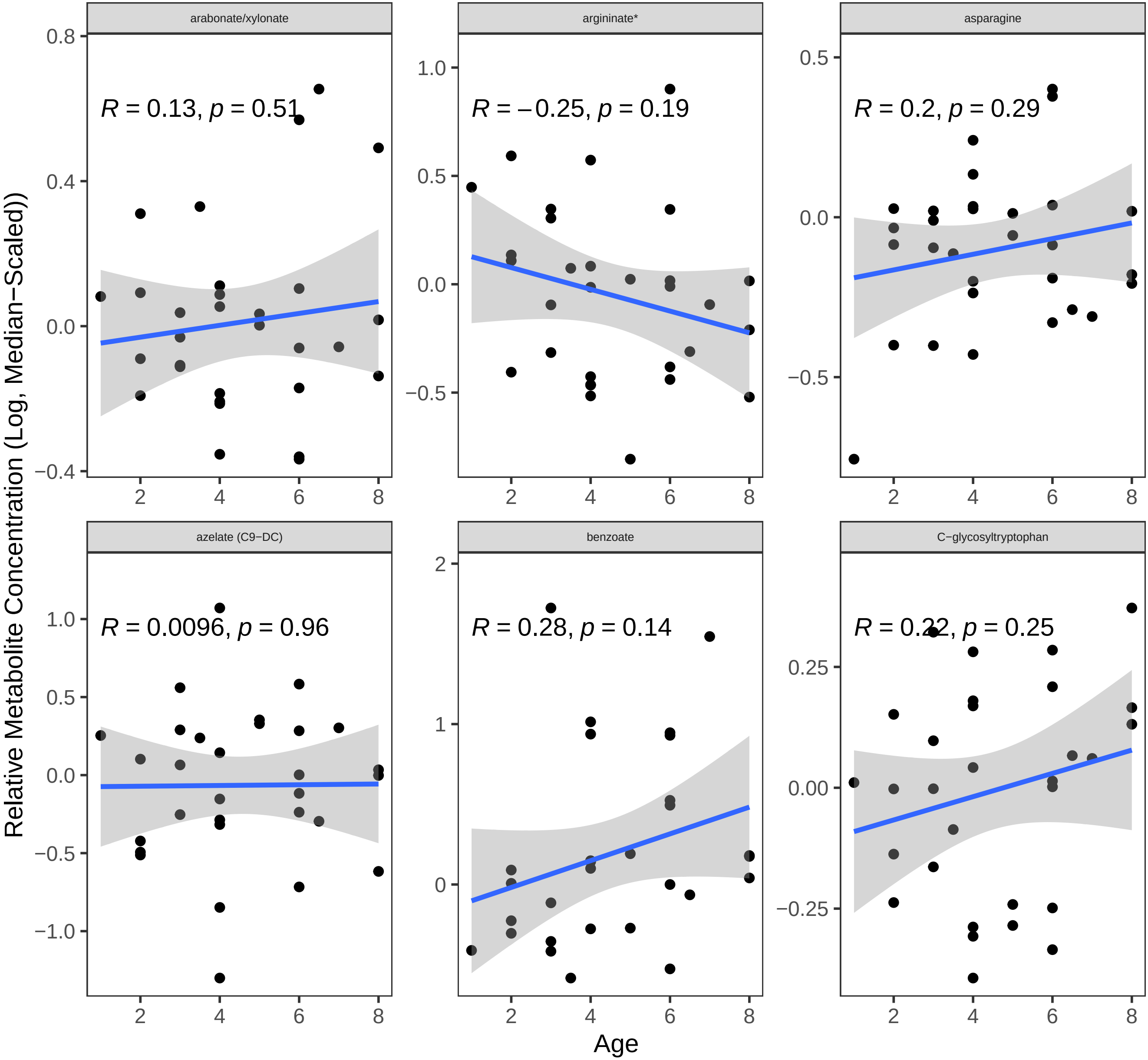
arabonate/xylonate
R = 0.13, p = 0.51
arargininate*
R = −0.25, p = 0.19
asparagine
R = 0.2, p = 0.29
azelate (C9−DC)
R = 0.0096, p = 0.96
benzoate
R = 0.28, p = 0.14
C−glycosyltryptophan
R = 0.22, p = 0.25
Relative Metabolite Concentration (Log, Median−Scaled))
Age

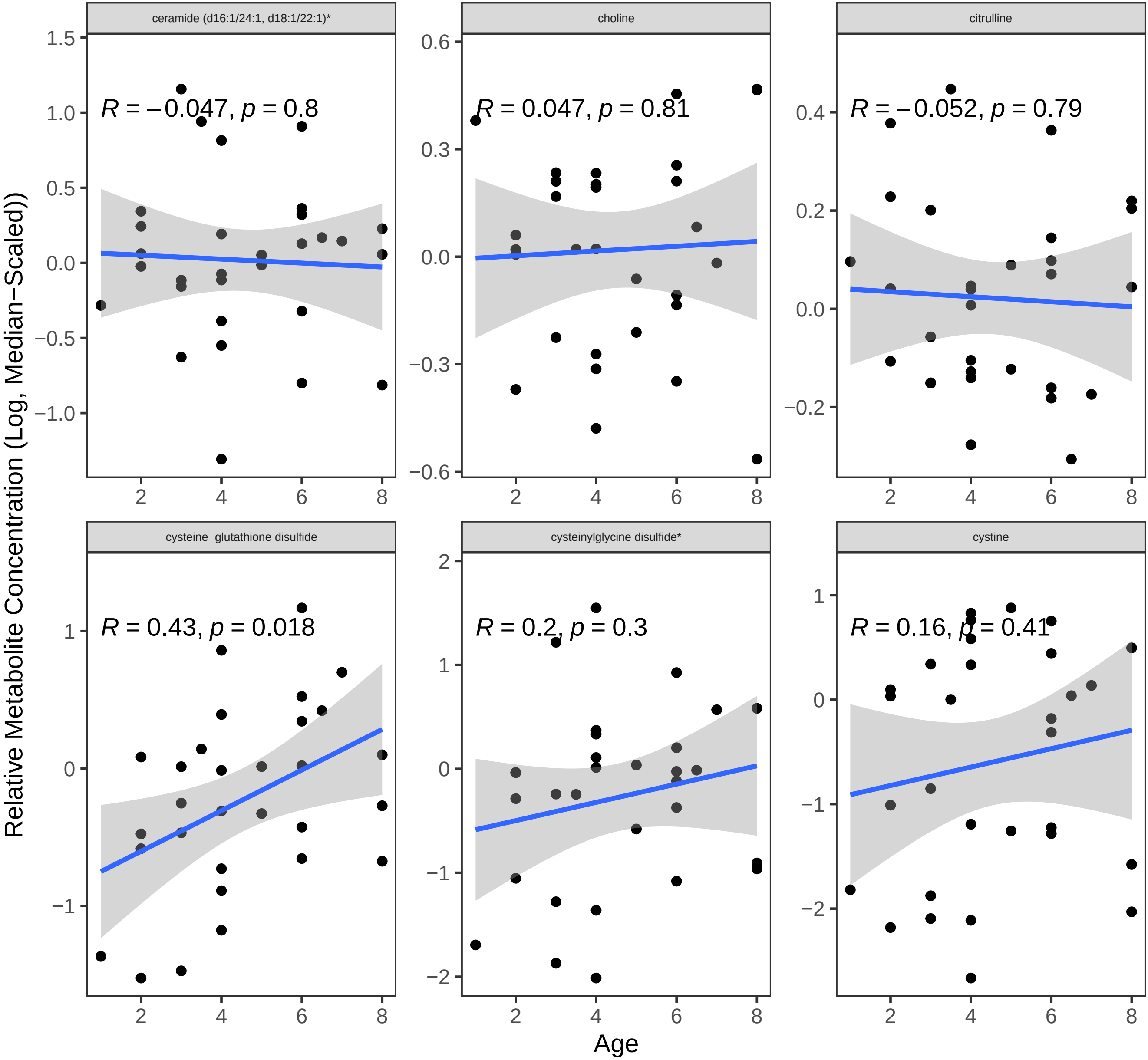
ceramide (d16:1/24:1, d18:1/22:1)*
R = −0.047, p = 0.8
choline
R = 0.047, p = 0.81
citrulline
R = −0.052, p = 0.79
cysteine−glutathione disulfide
R = 0.43, p = 0.018
cysteinylglycine disulfide*
R = 0.2, p = 0.3
cystine
R = 0.16, p = 0.41
Relative Metabolite Concentration (Log, Median−Scaled))
Age

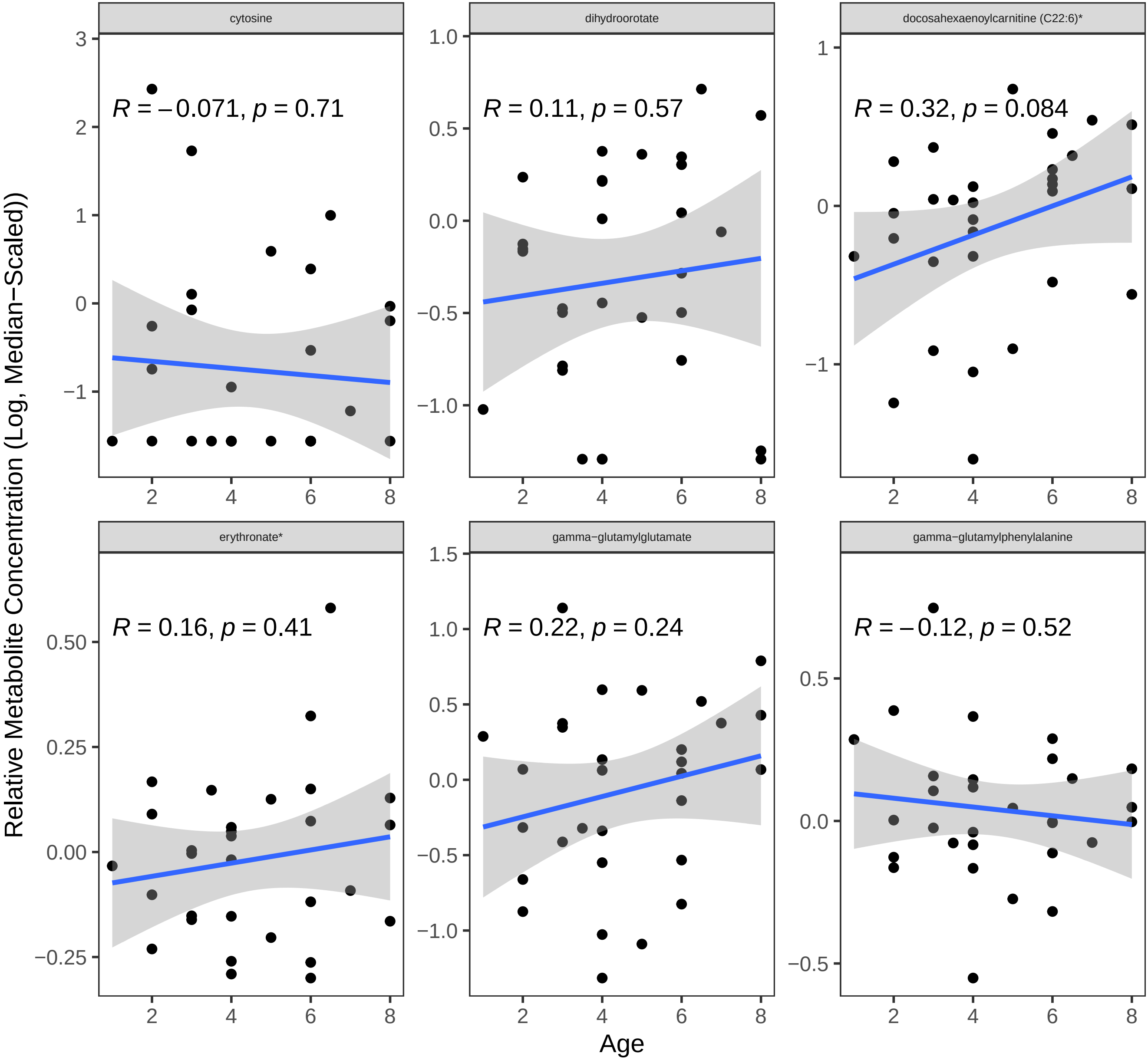
cytosine
R = −0.071, p = 0.71
dihydroorotate
R = 0.11, p = 0.57
docosahexaenoylcarnitine (C22:6)*
R = 0.32, p = 0.084
erythronate*
R = 0.16, p = 0.41
gamma−glutamylglutamate
R = 0.22, p = 0.24
gamma−glutamylphenylalanine
R = −0.12, p = 0.52
Relative Metabolite Concentration (Log, Median−Scaled))
Age

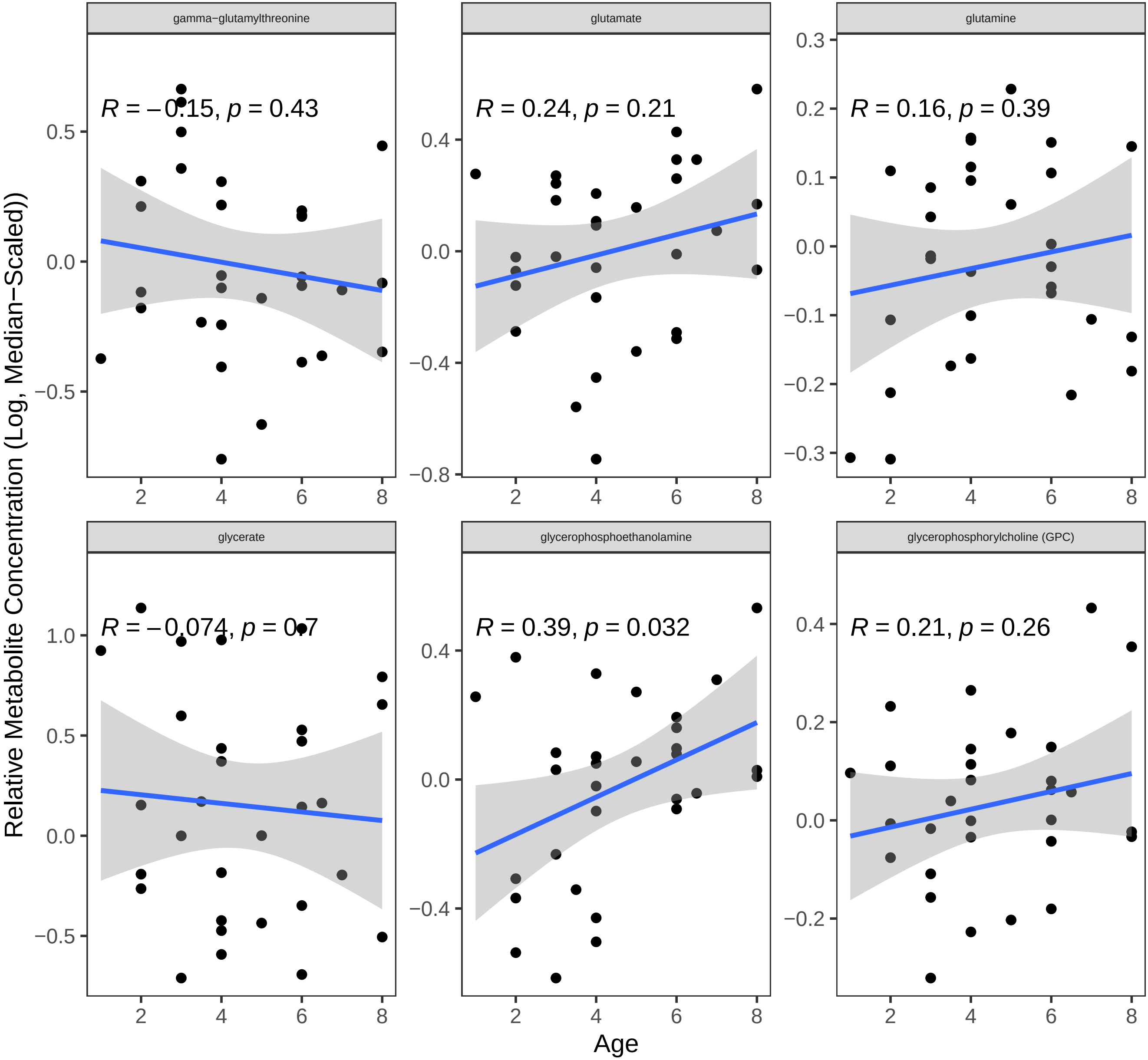
gamma−glutamylthreonine
R = −0.15, p = 0.43
glutamate
R = 0.24, p = 0.21
glutamine
R = 0.16, p = 0.39
glycerate
R = −0.074, p = 0.7
glycerophosphoethanolamine
R = 0.39, p = 0.032
glycerophosphorylcholine (GPC)
R = 0.21, p = 0.26
Relative Metabolite Concentration (Log, Median−Scaled))
Age

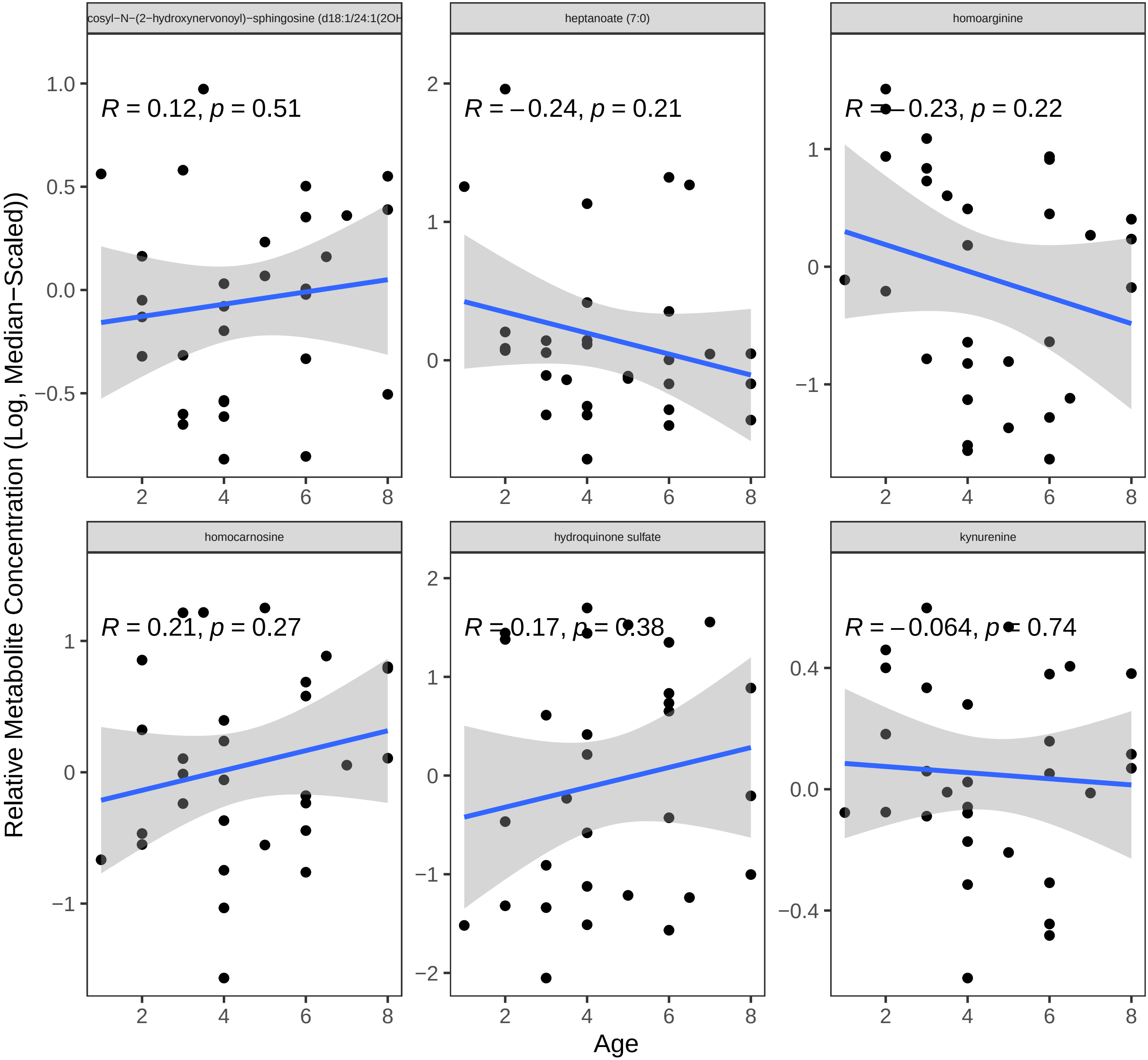

cosyl−N−(2−hydroxynervonoyl)−sphingosine (d18:1/24:1(2OH
$R = 0.12, p = 0.51$
heptanoate (7:0)
$R = -0.24, p = 0.21$
homoarginine
$R = -0.23, p = 0.22$
homocarnosine
$R = 0.21, p = 0.27$
hydroquinone sulfate
$R = 0.17, p = 0.38$
kynurenine
$R = -0.064, p = 0.74$
Relative Metabolite Concentration (Log, Median−Scaled))
Age

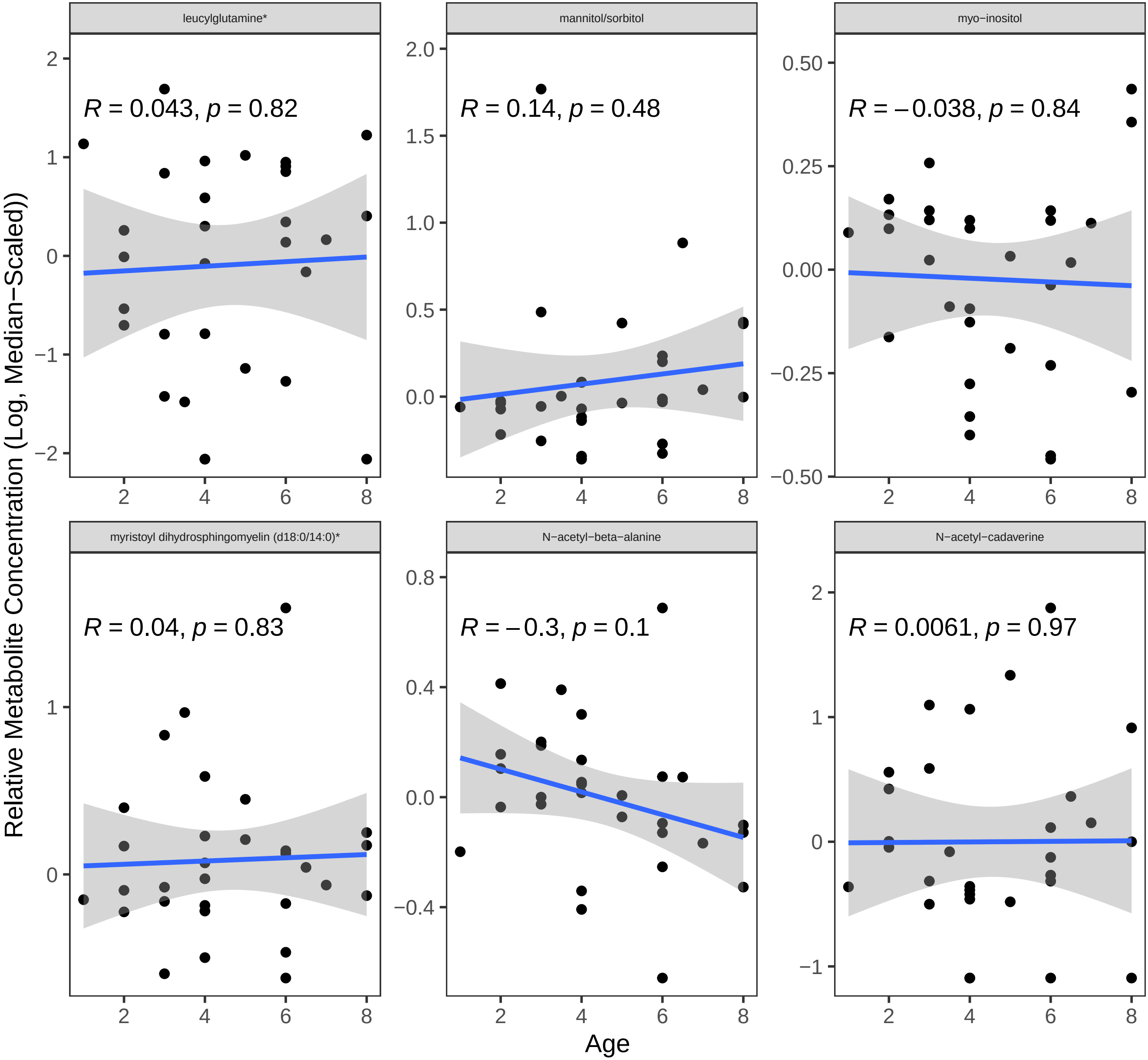
leucylglutamine*
R = 0.043, p = 0.82
mannitol/sorbitol
R = 0.14, p = 0.48
myo−inositol
R = −0.038, p = 0.84
myristoyl dihydrosphingomyelin (d18:0/14:0)*
R = 0.04, p = 0.83
N−acetyl−beta−alanine
R = −0.3, p = 0.1
N−acetyl−cadaverine
R = 0.0061, p = 0.97
Relative Metabolite Concentration (Log, Median−Scaled))
Age

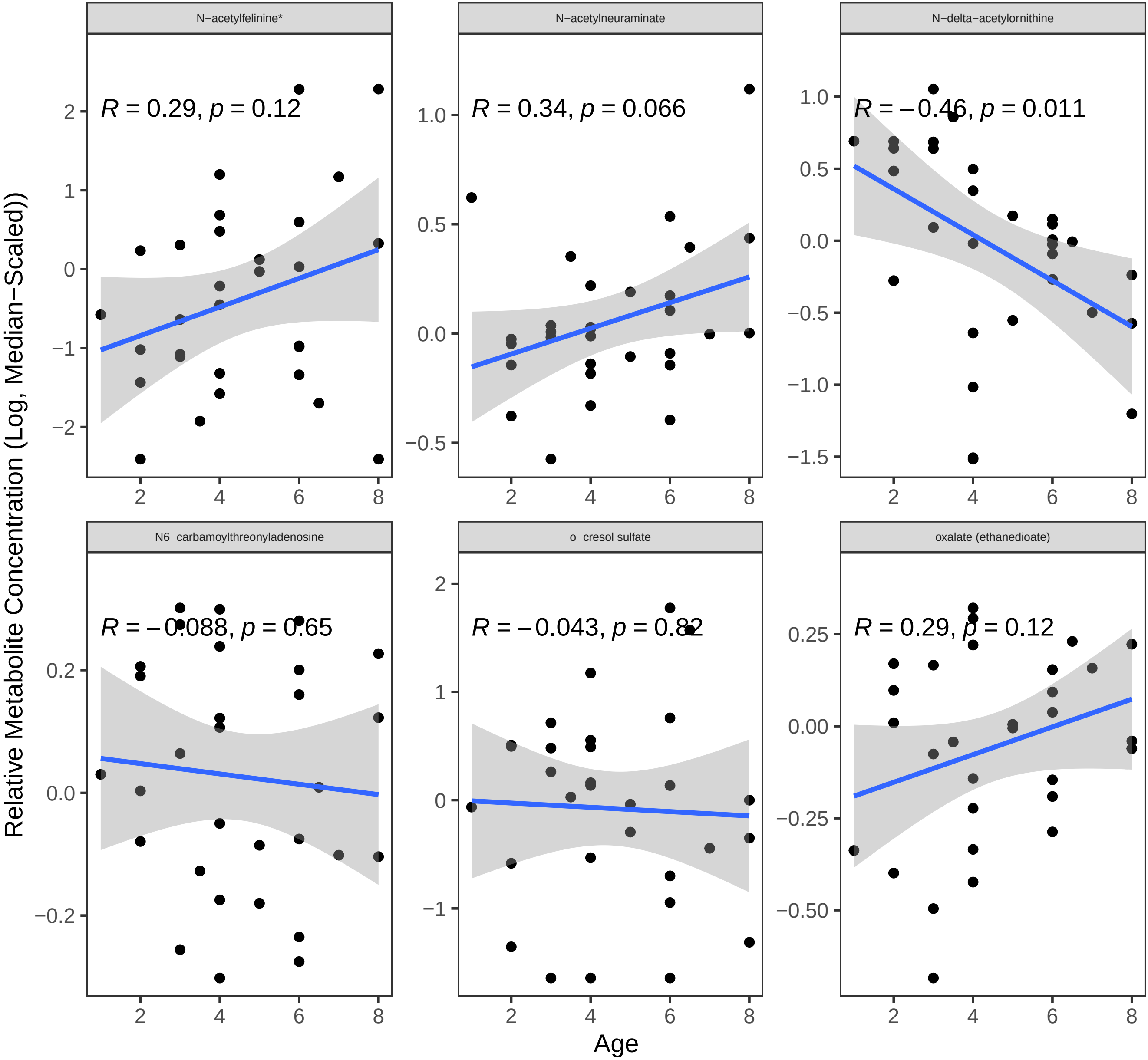
N−acetylfelinine*
R = 0.29, p = 0.12
N−acetylneuraminate
R = 0.34, p = 0.066
N−delta−acetylornithine
R = −0.46, p = 0.011
N6−carbamoylthreonyladenosine
R = −0.088, p = 0.65
o−cresol sulfate
R = −0.043, p = 0.82
oxalate (ethanedioate)
R = 0.29, p = 0.12
Relative Metabolite Concentration (Log, Median−Scaled))
Age

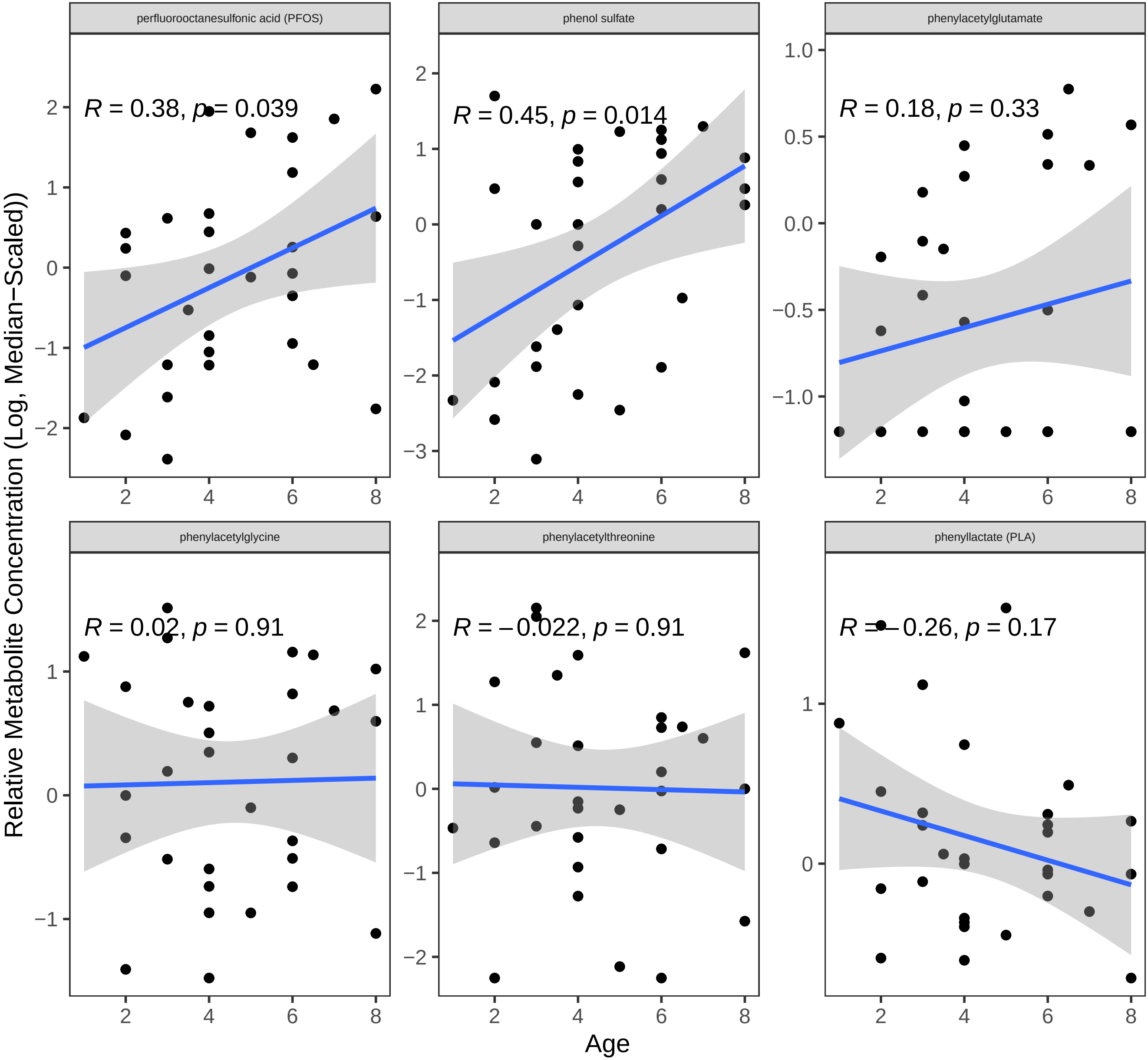
perfluorooctanesulfonic acid (PFOS)
R = 0.38, p = 0.039
phenol sulfate
R = 0.45, p = 0.014
phenylacetylglutamate
R = 0.18, p = 0.33
phenylacetylglycine
R = 0.02, p = 0.91
phenylacetylthreonine
R = −0.022, p = 0.91
phenyllactate (PLA)
R = −0.26, p = 0.17
Relative Metabolite Concentration (Log, Median−Scaled))
Age

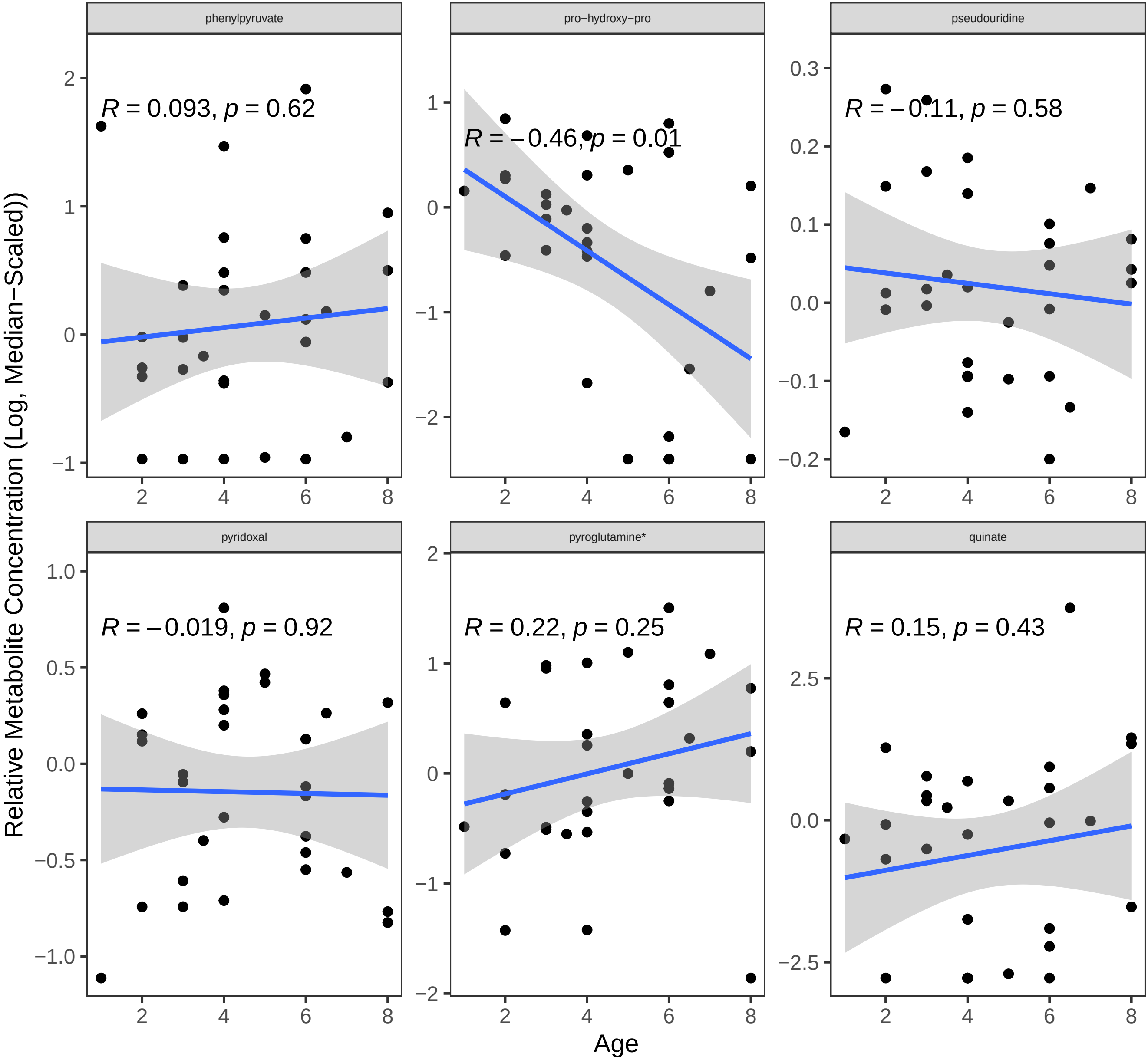

phenylpyruvate
R = 0.093, p = 0.62
pro−hydroxy−pro
R = −0.46, p = 0.01
pseudouridine
R = −0.11, p = 0.58
pyridoxal
R = −0.019, p = 0.92
pyroglutamine*
R = 0.22, p = 0.25
quinate
R = 0.15, p = 0.43
Relative Metabolite Concentration (Log, Median−Scaled))
Age

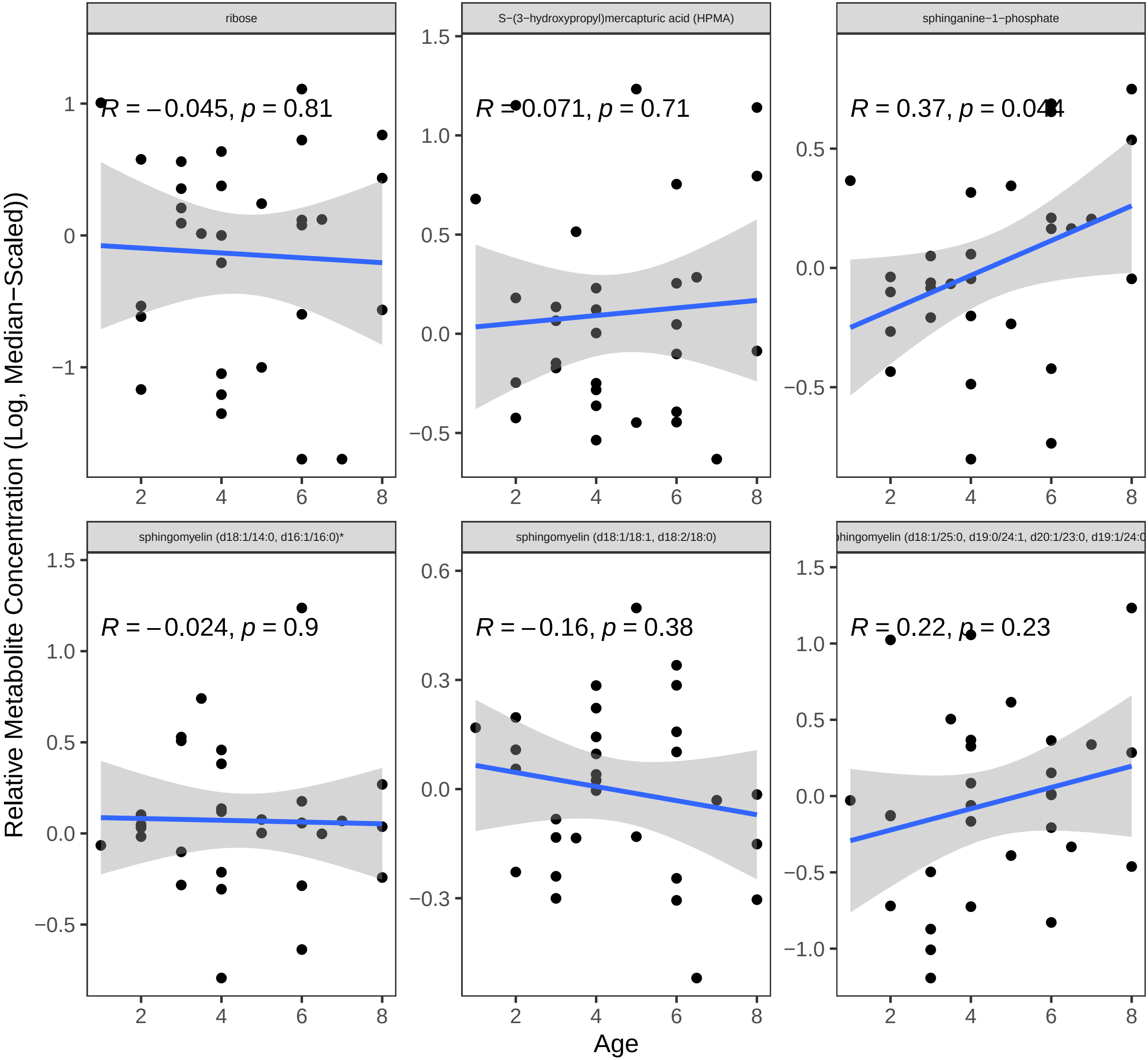
ribose
R = −0.045, p = 0.81
S−(3−hydroxypropyl)mercapturic acid (HPMA)
R = 0.071, p = 0.71
sphinganine−1−phosphate
R = 0.37, p = 0.044
sphingomyelin (d18:1/14:0, d16:1/16:0)*
R = −0.024, p = 0.9
sphingomyelin (d18:1/18:1, d18:2/18:0)
R = −0.16, p = 0.38
phingomyelin (d18:1/25:0, d19:0/24:1, d20:1/23:0, d19:1/24:0
R = 0.22, p = 0.23
Relative Metabolite Concentration (Log, Median−Scaled))
Age

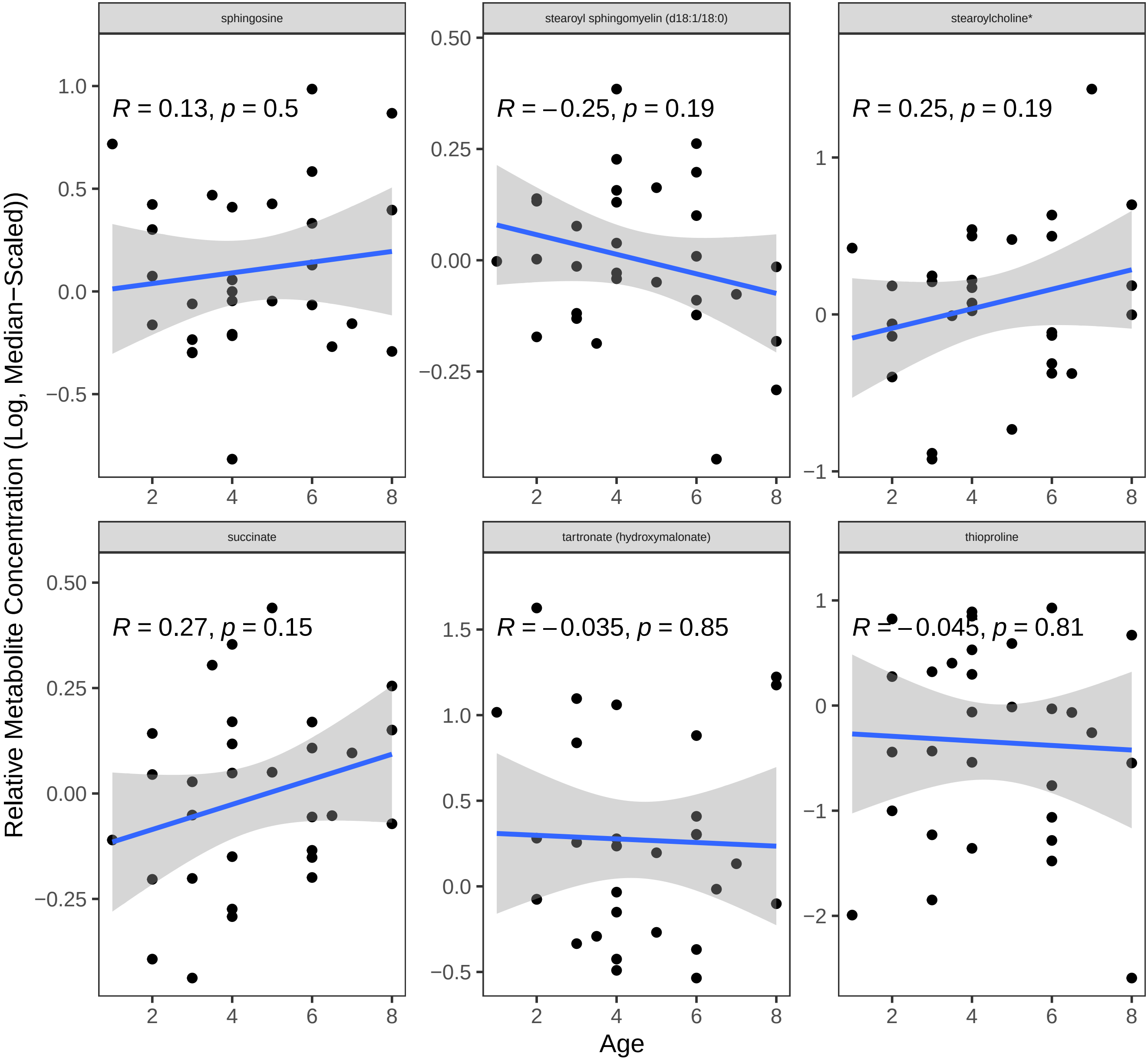
sphingosine
R = 0.13, p = 0.5
stearoyl sphingomyelin (d18:1/18:0)
R = −0.25, p = 0.19
stearoylcholine*
R = 0.25, p = 0.19
succinate
R = 0.27, p = 0.15
tartronate (hydroxymalonate)
R = −0.035, p = 0.85
thioproline
R = −0.045, p = 0.81
Relative Metabolite Concentration (Log, Median−Scaled))
Age

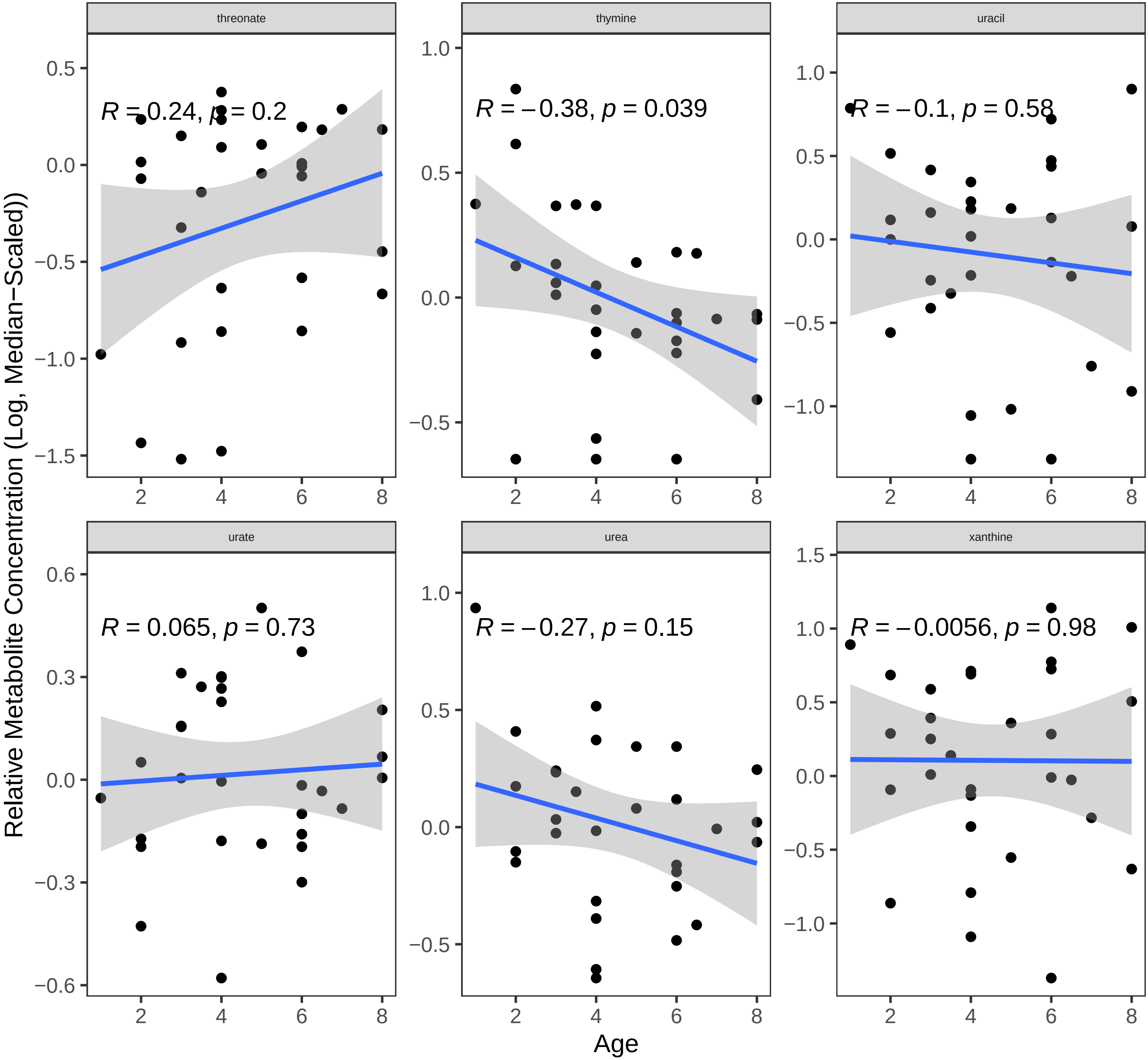
threonate
thymine
uracil
urate
urea
xanthine
R = 0.24, p = 0.2
R = −0.38, p = 0.039
R = −0.1, p = 0.58
R = 0.065, p = 0.73
R = −0.27, p = 0.15
R = −0.0056, p = 0.98
Relative Metabolite Concentration (Log, Median−Scaled))
Age
