# Supplementary material for: Untargeted Analysis of Serum Metabolomes in Dogs with Exocrine Pancreatic Insufficiency
Source: Animals (Basel). 2023 Jul 14;13(14):2313. doi: 10.3390/ani13142313 (PMC10376357; doi:10.3390/ani13142313)
Supplement: Supplementary file 1 [file animals-13-02313-s001.zip › animals-2380457-supplementary/Supplemental Info/File S6.pdf]

Untargeted analysis of serum metabolomes in dogs with exocrine pancreatic insufficiency

Supplementary Information

Serum metabolite profiles were compared in healthy dogs before (PRE) and after (POST) 14 days of oral supplementation with oral pancreatic enzyme extracts (porcine pancreatin 6X).

1) Statistical heatmap of Euclidian distances of relative metabolite abundances.

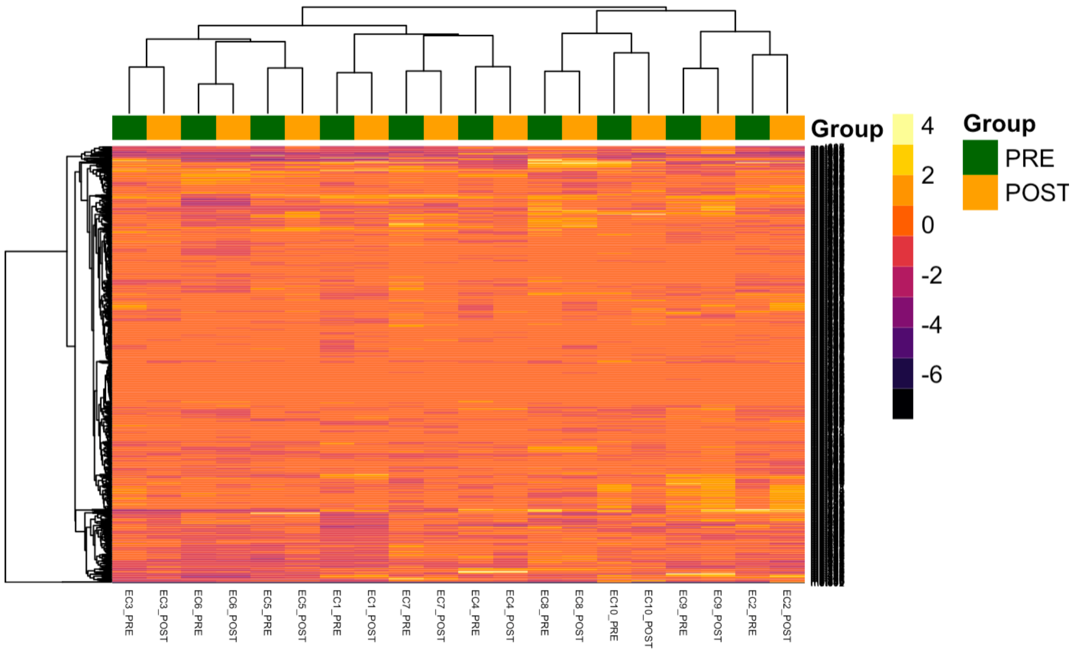

2) Principal component analysis:

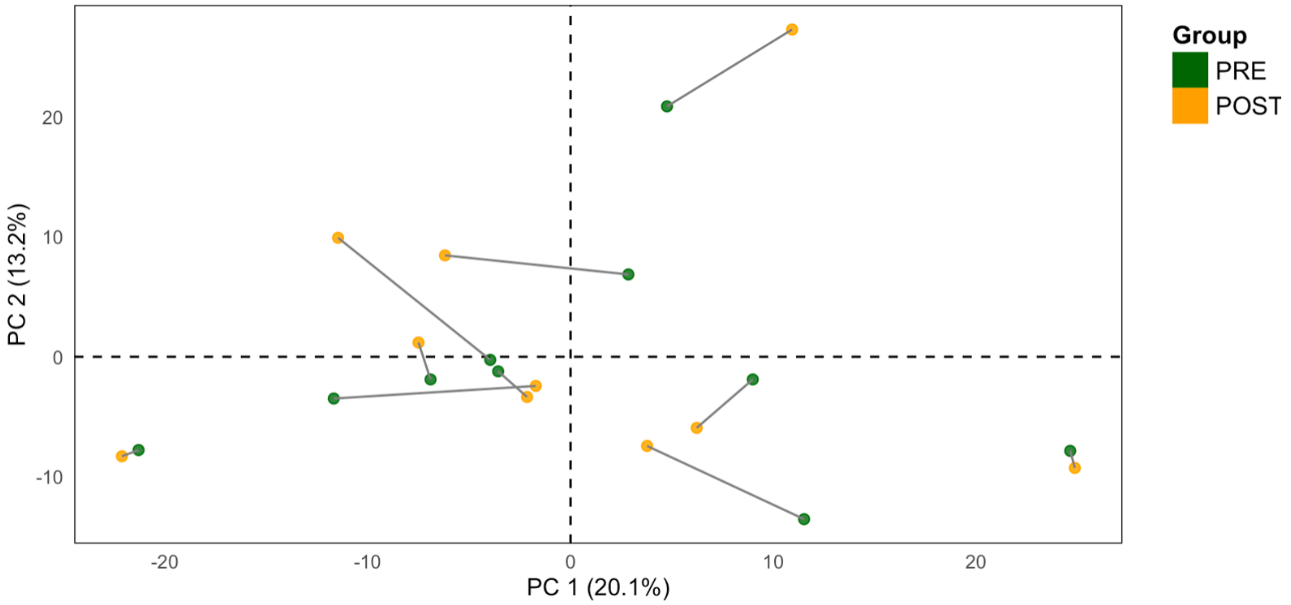

Untargeted analysis of serum metabolomes in dogs with exocrine pancreatic insufficiency

Supplementary Information

3) Differential Analysis of Relative Metabolite Abundances

Paired t-tests were used on log-transformed relative metabolite abundances and the p-values were corrected for multiple testing using the Benjamini-Hochburg false discovery rate method (FDR, q-values).

After controlling the FDR at q<0.2, there were no serum metabolites that varied significantly after pancreatic enzyme supplementation in healthy dogs.

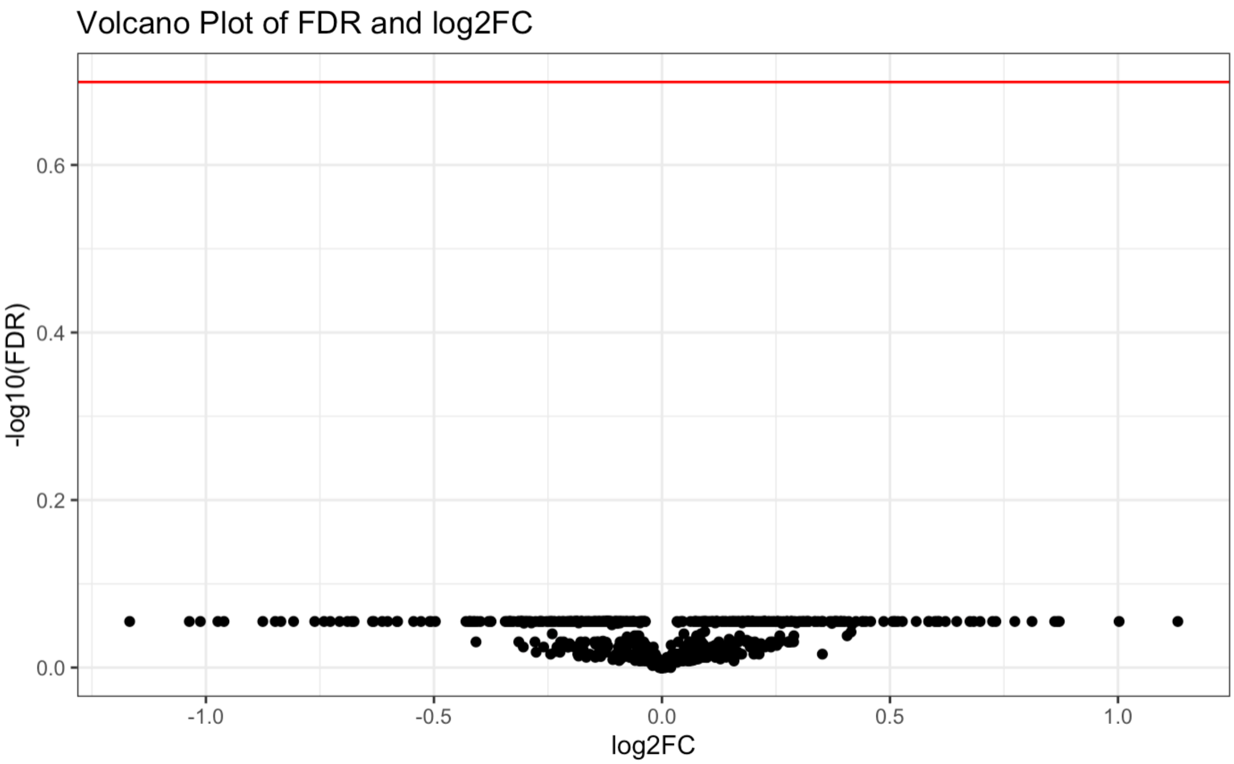

The red line represents the threshold for statistical significance at FDR (q-value)<0.2.
